# Transcriptomic data and biomedical literature synergize in finding pharmacologic gene regulators

**DOI:** 10.64898/2026.03.13.708862

**Published:** 2026-03-14

**Authors:** Cole A. Deisseroth, Bess Brazelton, Zahid Shaik, Zhandong Liu, Huda Y. Zoghbi

**Affiliations:** 1 Medical Scientist Training Program, Baylor College of Medicine, Houston, TX, 77030, United States; 2 Jan and Dan Duncan Neurological Research Institute, Houston, TX, 77030, United States; 3 Genetics and Genomics Graduate Program, Baylor College of Medicine, Houston, TX, 77030, United States; 4 Department of Molecular and Human Genetics, Baylor College of Medicine, Houston, TX, 77030, United States; 5 Department of Pediatrics, Baylor College of Medicine, Houston, TX 77030, United States; 6 Department of Neuroscience, Baylor College of Medicine, Houston, TX 77030, United States; 7 Howard Hughes Medical Institute, Chevy Chase, MD 20815, United States

## Abstract

Most Mendelian disorders caused by a deficiency or excess of one gene product lack targeted therapies. Since these disorders can be modeled with a gene overexpression, knockout, or knockdown, drugs that oppose the transcriptomic effects of such perturbations may be promising therapeutic candidates. RNA-Sequencing (RNA-Seq) studies can fuel this drug-prioritization, but their labels, written in plain language, must be annotated manually. Hence, we introduce Signature-based Networks from Automatically Curated Knockout, Knockdown, and Small-molecule Studies (SNACKKSS), which automatically curates gene-disruption and drug studies from the Gene Expression Omnibus and, in partnership with uniformly computed read count datasets, feeds the labels and RNA-Seq data directly into regulatory relationship predictions. Through cross-validation, we show that SNACKKSS’ predictions (specifically, from a variation called “SA4”) make a unique contribution to finding protein-inhibiting compounds, even alongside existing predictors. We demonstrate the benefit of aggregating multiple predictive tools, and provide this powerful ensemble alongside SNACKKSS. Importantly, we advise researchers to test complex machine learning models on multiple devices. Even with code packages kept consistent, they can run deterministically within a machine, but inconsistently on different ones. Nonetheless, the downstream predictive ability was striking, and leveraging multiple sources of information, RNA-Seq data included, will vastly improve drug-repurposing screens.

## Introduction:

In the field of medical genetics, tremendous advances have been made in our ability to workup(1), diagnose(2), and even treat(3) individuals presenting with rare Mendelian disorders. One would ideally treat the underlying cause, as opposed to simply managing symptoms. Targeted gene therapies (ongoing efforts reviewed by Laurent et al(4)) and engineered molecules that specifically address the defect(3, 5) are in active development, with some now having Food and Drug Administration (FDA) approval(6). Another approach is drug repurposing—finding chemicals with well-established toxicity profiles, originally intended for a different disorder, that also happen to regulate the affected gene product in a Mendelian disorder. General examples are reviewed by Sun et al(7), and Clement Chow’s lab has been running repurposing screens in fruit flies, to find treatments for congenital disorders of glycosylation(8, 9). If there is a molecule with a good safety profile that is particularly likely to address the deficiency or excess of a given gene product, it could be tested and brought to the bedside quickly(7). Therefore, a bioinformatic tool that can reliably identify the most promising drugs to test could save time and resources in these screens, and expedite the treatment of rare disorders. Many have tried to build such a tool, using automated literature-parsing pipelines(10–12) and pathway-analysis algorithms that rely on authors’ interpretation of their own data, but there is increasing interest in approaches that instead utilize the data directly. RummaGene(13) parses data tables in the medical literature, as opposed to the main text, and links genes together based on their frequency of co-occurrence; All RNA-Seq and ChIP-Seq Sample and Signature Search (ARCHS4)(14) provides not only a database of uniformly computed read counts from the RNA-Sequencing (RNA-Seq) samples in GEO, but also correlations between two genes’ expression levels across human and mouse samples; and RummaGEO(15) automatically groups samples within each RNA-Seq study in ARCHS4’s read count database, calculates differential expression, and links genes together based on their frequency of co-occurrence in differentially expressed gene (DEG) lists. What these approaches have in common, alongside other correlative measures such as CoRegNet(16), gene set enrichment analysis(17), and the weighted gene co-expression network analysis(18), is that they do not establish temporality, and thus cannot distinguish causation from correlation.

In one attempt to address this deficit, RummaGEO was paired with known transcription-factor binding sites to infer this causation for transcription factors, in the ChEA-KG resource(19), formerly known as hGRN-ChEAR(20). Another attempt was the Connectivity Map (CMap)(21), which provides this information on temporality, in a massive screen of gene-perturbations and drug treatments in numerous human cell lines, using a microarray targeting 978 genes. CMap’s flagship study found molecules with similar effects on the transcriptome to the knockouts of specific tyrosine kinases, and experimentally verified that those molecules were binding to and inhibiting those kinases(21). It was unclear how many false predictions they had to sift through before they settled on these correct ones, and augmenting signatures over 10-fold from a small microarray might not be an adequate substitute for RNA-Seq (22). Still, the data have shown considerable promise in guiding drug-repurposing efforts, as Rachel Melamed’s lab showed a strong correlation between similarity of drugs’ differential gene expression profiles (or transcriptomic “signatures”) and their propensity to have the same therapeutic indications(23). This approach has considerable potential in optimizing treatment regimens for common health conditions, but for rare Mendelian diseases with no known therapy, it is unknown how (or whether) transcriptomic data can effectively guide the search for an effective treatment.

Thanks to data-sharing policies and centralized repositories such as the Gene Expression Omnibus (GEO)(24), RNA-Seq has become one of the most abundant omic data modalities. It has previously been proposed to match signatures of drug treatments and either disease states(25–27) or gene disruptions(25) to predict the therapeutic benefit of the given drug, but such approaches need to be scalable to the millions of samples in GEO. To make GEO-wide analyses more feasible, multiple labs ran uniform alignment on the raw reads of most of the human and mouse samples in the Sequence Read Archive (SRA, which is tied to the metadata in GEO) to convert them into gene (or transcript) read counts. This resulted in databases such as ARCHS4(14), Recount3(28), and Digital Expression Explorer 2 (DEE2)(29), which together lowered a tremendous barrier in the re-use of these data.

The remaining challenge, however, is identifying the experiments that were done and which samples were in which group. Descriptions of such are provided by GEO in plain language, but meta-analyzing numerous studies from different authors requires a means of encoding all of the different annotation formats into one. Artificial intelligence (AI) has previously been used to systematically encode the study descriptions in GEO, but none quite capture what we need in order to automatically collate gene-disruption and drug experiments for a meta-analysis. Meta-SRA(30) uses a rule-based text-reasoning graph to extract key study information, but is no longer maintained as of 2020. GEOracle(31) uses a support vector machine to separate samples, and GeMI(32) uses a transformer model, but neither tool indicates what perturbagen distinguishes one group from the other, and the latter is no longer available. Lastly, the Crowd Extracted Expression of Differential Signatures (CREEDS) tool(33) uses crowd-sourced manual annotations of microarray studies in GEO (including what gene was being perturbed, what drug was administered, and which samples were in the experimental and control groups) to train a classifier to provide these annotations for other studies. After the classifier was run, the training data (which are reusable) and the predictions that it made on other studies were both made publicly available. However, it is not being updated; it was only trained and run on microarray studies; and the participants in the crowd-sourced data curation annotated whichever studies they wanted to (so we presume, without their paper stating the contrary), which may bias the dataset in favor of the studies that are easy to interpret. The last point is not necessarily a drawback, as one could argue that the ambiguously annotated studies can add noise and should thus be avoided; but it is still worthwhile to attempt to salvage them.

Our aim is to fully operationalize the annotation of publicly available transcriptomic studies on drug-treatments and gene-disruptions, and determine whether these automatically curated studies can drive reliable regulatory relationship predictions that other tools would have missed. Hence, we introduce Signature-based Networks from Automatically Curated Knockout, Knockdown, and Small-molecule Studies (SNACKKSS), which uses a series of fine-tuned Bidirectional Encoder Representations from Transformers (BERT) models(34) to annotate the GEO metadata, and feeds these annotations directly into a signature-matching and relationship-prediction pipeline without any manual intervention. With this pipeline in place, we then test its ability to prioritize correct modulators over incorrect ones, and the contribution that it makes alongside existing predictive tools. Lastly, we demonstrate the vastly improved predictive ability achieved by an ensemble of many predictive tools (including a variant of SNACKKSS), which will be available for download at snackkss.nrihub.org upon submission for peer review.

## Materials and Methods:

### Reagents; Biological Resources: Not applicable

#### Novel Programs, Software, Algorithms:

The code used to run the analyses in this article is divided into three publicly available GitHub repositories: https://github.com/coledeisseroth/SNACKKSS_NLP, https://github.com/coledeisseroth/SNACKKSS, and https://github.com/coledeisseroth/SNACKKSS_Eval. The latest study curations and corresponding relationship predictions will be available at snackkss.nrihub.org upon submission for peer review.

#### Web Sites/Data Base Referencing:

Gene Expression Omnibus(24), from no later than July 24, 2025.

CREEDS(33), accessed on December 16, 2023.

ARCHS4’s read counts(14), version 2.5.

ARCHS4’s correlations(14), version 2.4.

Recount3’s read counts(28) on July 30, 2025.

DEE2’s read counts(29), accessed from July 31 to August 1, 2025.

PARMESAN(12), corpus timestamped on February 15, 2025.

Connectivity Map(21), level-5 MODZ scores, downloaded on July 20, 2025, but some of the data files are timestamped at December 16–17, 2020.

DGIdb(35), version from December 6, 2025.

Reactome’s functional interaction dataset(36), version from April 14, 2025.

DIOPT(37), accessed July 25, 2025.

Entrez gene database (https://www.ncbi.nlm.nih.gov/gene/), accessed July 20, 2025.

PubChem (https://pubchem.ncbi.nlm.nih.gov/) Substance IDs, accessed July 19, 2025.

### Random selection of high-throughput sequencing series

To ensure an unbiased sampling of GEO studies, we queried GEO on May 20, 2024 for all series IDs under the label “Expression profiling by high-throughput sequencing”, of which there were 97,822. We shuffled them with a random seed of “2024”. C.A.D., a predoctoral trainee, annotated the first 10 of these series, indicating any relevant genetic or pharmacologic perturbations and how to distinguish control from experimental samples. Under his supervision, a high-school-level researcher, B.B., annotated another 615 during a three-week internship. C.A.D. subsequently made corrections in order to ensure that all of the annotated studies were accounted for (either fully processed or recognized as not processable) by the scripts that were processing them—these scripts would be reading “soft” files instead of the GEO web portal that the manual curators were using. He also changed any labels that he disagreed with. For transparency, we provide both the uncorrected and the corrected versions of this manually curated dataset (Supplemental tables 1 and 2, respectively), and for the remainder of this study, we only use the corrected version. We call our manually curated dataset “SNACKKSS-Manual Curation”, or “SNACKKSS-MC”, and display in Supplemental Figure 1 the number of GEO series with each type of perturbation in it and in CREEDS’ dataset. Details on the incorporation of CREEDS’ dataset are provided in Supplemental methods.

### BERT models were trained on manual annotations

Our processing pipeline had four key steps: (1) Study classification: whether the study is testing a gene disruption; (2) Sample classification: whether a given sample is receiving a gene disruption; (3) Target classification: what gene was disrupted; (4) Control classification: given one sample that had a specific perturbation and one that did not, whether the latter is an appropriate control to the former. For example, if there are multiple cell lines tested, then the controls for a given perturbation should come from the same line. Drug-treatment studies are curated analogously to this.

We tested three BERT(34) models on each of these tasks: DistilBERT(38), a smaller BERT model trained to mimic the original one; BioBERT(39), the original BERT model, fine-tuned on medical literature; and BioMedBERT(40), the original BERT model trained exclusively on the medical literature, from default weights instead of BERT’s pre-trained ones. For each model, we would measure the precision, recall, and F1 score in each task. We used 4-fold cross-validation (See Supplemental table 3 for the series IDs put into each group) to train and test each model on both our manually curated dataset, and the one from CREEDS. We also tested the performance from training each model on CREEDS’ dataset followed by ours, as well as the reverse. Because our manually curated dataset is a random selection of high-throughput sequencing studies, instead of human-selected microarrays, we used the model that performed the best on our dataset, even if another model performed better on CREEDS’.

Precision is calculated as true positives / (true positives + false positives + 1). Recall is true positives / (true positives + false negatives + 1). The F1 score is calculated as 3 * precision * recall / (precision + recall + 1). The denominators are incremented by 1 to penalize smaller sample sizes, and the F1 score formula is adjusted so that it remains scaled from 0 to 1. Further details on input-formatting, accuracy testing, and synonym resolution are provided in the Supplemental methods.

### The best fine-tuned model is identified for each classification task

For each task—classifying studies, disruption samples, targets, and then controls—we test each BERT model after training it on either our dataset (SNACKKSS-MC), CREEDS’ dataset, SNACKKSS-MC and then CREEDS’, or CREEDS’ and then SNACKKSS-MC. 4-fold cross-validation (See Supplemental methods) allows us to test each fine-tuned model on the full dataset. We test the performance on both CREEDS’ dataset and SNACKKSS-MC for transparency, but we only use SNACKKSS-MC for deciding which fine-tuned model we ultimately use. We run the best study classifier on all of the human and mouse RNA-Seq study descriptions that we were able to acquire; then the best sample classifier on the sample descriptions from all studies labeled as positive by the study classifier; then the best target classifier on the descriptions of all samples labeled as positive. We then run the control classifier on the samples with automatically identified targets. Control classification is more computationally intensive than the other steps, because we are classifying pairs of samples, of which there can be millions in a single study. We thus apply five filters to pairs (see Supplemental methods) to make the job feasible for our machines.

### Perturbation signatures are calculated across curated experiments

Because different studies can use different RNA-Seq alignment tools to establish read counts for each gene, the standard approach to RNA-Seq meta-analyses is to re-align the raw reads, using a uniform alignment pipeline(14, 28, 29, 41). This can be a trivial task for two or three studies, but when conducting large-scale meta-analyses across hundreds of thousands of samples, the computational cost of alignment requires dedicated computational resources. Multiple research teams have taken on this role, generating publicly available resources such as ARCHS4(14), Recount3(28), and DEE2(29), making it cost-effective and reproducible to calculate signatures for the tens of thousands of gene-disruption and drug-studies. In each of the three pre-computed read count datasets, ARCHS4, Recount3, and DEE2 (specifically, DEE2’s Kallisto(42) expression files), we procure any available read counts for that sample and its controls. Our approach to handling samples from different read count datasets is described in Supplemental methods.

Having normalized each sample to its respective controls, we next need to establish overall differential gene expression patterns across samples that had the same gene disrupted. There are many established ways to collapse signatures into unified consensuses—CMap uses a “MODZ” score consisting of a weighted average of expression z-scores in perturbed samples(21), and the authors of L2S2 tested multiple consensus approaches for their ability to detect CMap drug signatures that are consistent with manually curated signatures from GEO(22). Future work may include finding the optimal collapsing method for finding regulatory relationships. For this study, however, we simply calculate the z-score of the perturbed samples’ z-scores, which should suffice if signature-matching is a valid predictive measure. For a disrupted gene A, we calculate the mean and standard deviation of the z-scores of a given gene B across all of the samples that had gene A disrupted. We then calculate a z-score from this distribution, thus giving one unified estimate of the effect of gene A disruption on gene B expression.

### Perturbation signatures are matched to predict regulatory relationships

The remaining question is whether the correlation between the signatures of two perturbations is suggestive of their relationship. Theoretically, two genes with similar disruption signatures would play roles supporting each other(21), whereas two genes whose disruptions have opposite effects on the transcriptome would have functions that normally oppose each other. Likewise, a small molecule whose administration yields similar or opposite effects to a gene’s disruption would oppose or support that gene’s function, respectively. To match signatures, we use a formula of our own design, which we give the moniker “Differential F1” or “DF1”. This formula is commutative, and can be run at high throughput in Bash. There are numerous existing methods for signature-matching, such as the weighted connectivity score(21), or simply a Pearson or Spearman correlation (both of which have been used in the context of co-expression(14, 43), and the latter of which has been used for matching drug signatures(23)). We will not speculate on which is the most appropriate, as this can only be decided by downstream predictive ability. Future work will include finding the optimal signature-matching method, but we use DF1 for a high-speed proof of concept due to the number of signature pairs that need to be matched in this study. If signature matching is a viable relationship-prediction method, DF1 should be sufficient, even if it is not optimal.

DF1 is calculated as follows, for matching the signatures of Perturbations A and B: we first set a threshold T, where we will only accept a differentially expressed gene (DEG) if the absolute value of its nested z-score is larger than T. We then determine the ability of A’s signature to “detect” the DEGs in B’s signature, and quantify this with an F1-like score. Let STP (supportive true positives) = the number of DEGs shared by A and B in the same direction (i.e. both z-scores are the same sign), OTP (opposing true positives) = the number of shared DEGs where A and B yielded opposite directions, FP (false positives) = the number of DEGs in A’s signature but not B’s, and FN (false negatives) = the number of DEGs in B’s signature but not A’s. We then calculate supportive and opposing precision (SP and OP, respectively) as:

(1) $$SP=\frac{STP}{STP\;+\;FP\;+\;OTP\;+\;1};OP=\frac{OTP}{OTP\;+\;FP\;+\;STP\;+\;1}$$

Likewise, we calculate supportive and opposing recall (SR and OR, respectively) as:

(2) $$SR=\frac{STP}{STP\;+\;FN\;+\;1};OR=\frac{OTP}{OTP\;+\;FN\;+\;1}$$

And we calculate the supportive and opposing F1 scores (SF1 and OF1, respectively) as:

(3) $$SF1=\frac{3\;*\;SP\;*\;SR}{SP\;+\;SR\;+\;1};OF1=\frac{3\;*\;OP\;*\;OR}{OP\;+\;OR\;+\;1}$$

Our final directionality score, like the one used by PARMESAN(12), penalizes the presence of conflicting information in both directions:

(4) $$DF1=\frac{2\;*\;(SF1\;-\;OF1)\;*\;|SF1\;-\;OF1|}{SF1\;+\;OF1\;+\;1}$$

The 1s added to the denominators are designed to penalize instances where there are few genes in a signature.

We then set the threshold T through leave-one-out cross-validation (LOOCV): we calculate all DF1 matches with values of 0, 0.1, 0.2… up to 0.9. For each relation in the manually curated dataset from modifier A to target B, we determine which of the ten thresholds is the most effective at prioritizing correct predictions over incorrect ones (highest log-rank test statistic) among the relations involving neither A nor any of its targets. Using that threshold, we then determine the DF1 score between A and B. We run separate LOOCV optimizations for positive and negative predictions, and eliminate any predictions where the directionality is different between the two optima. In other words, for a given two entities, if the positive LOOCV optimization led to a positive prediction score, and the negative optimization led to a negative one (or vice versa), the relationship between those two entities will not be predicted.

### Accuracy testing uses open-source methods

All denominators in our accuracy tests are incremented by 1 to penalize small sample sizes—for example, if there is only one prediction and it was correct, the accuracy will be reported as 1 / 2.

In our original publication on PARMESAN(12), we measured the total number of regulatory relationships predicted at the smallest score achieving each directional accuracy. The predictive measures we introduce in this study, however, are far more computationally intensive, requiring us to limit our evaluation to the regulatory relations that we can verify with DGIdb or Reactome. This is a less-informative measure of the absolute coverage of these tools, but it is still effective for comparing different methods.

Additionally, in contrast to said publication, we have begun to eschew our reliance on the paid black-box statistical tools in GraphPad Prism, so that we could have our accuracy evaluations and hypothesis tests as a part of our open-source pipeline (or in Microsoft Excel, as needed). For evaluation of one predictor’s ability to prioritize correct relations over incorrect, we transitioned from Prism’s extra sum-of-squares F test (comparing the one-phase-decay constants of correct and incorrect as we increase the score threshold) to a log-rank test (still comparing the decay rates of correct and incorrect predictions as we increase the score threshold). For comparing the coverage of different prediction tools, we transitioned from a Friedman rank-sum test measuring coverage at integer-percent accuracy thresholds to a binomial test for the proportion of predictor A’s achieved accuracy levels where predictor B achieved a larger number of predictions at a greater accuracy. Granted, if a user knows what accuracy level they want, then a statistical test that measures superiority across accuracy levels will not be helpful.

Furthermore, because we are testing several permutations of a default pipeline for their ability to prioritize correct over incorrect, we apply Bonferroni correction to our log-rank p-values. Even though we unconditionally adhere to our default setup, we evaluate each permuted pipeline for whether it can prioritize correct over incorrect. Any of them succeeding would theoretically be considered a positive result, and the Bonferroni correction must therefore account for all of them. There are 72 total signature-based predictors we are evaluating: 2 (direct-matching and linking to ARCHS4) * 2 (supportive and inhibitory) * 2 (gene-gene and drug-gene relations) * 9 (4 permutations of SNACKKSSS and 5 of CMap). Hence, we multiply all log-rank p-values by 72 to get the adjusted p-value. Importantly, we do not account for the p-values from the log-rank tests at each of the ten thresholds, because we would not consider it a success if one of them had a strong prioritization ability.

### SNACKKSS is permuted to assess whether the chosen features are optimal

One could argue that any failure in SNACKKSS’ predictive ability is due to poorly conducted signature-matching, rather than inherently poor performance of signature-matching. Therefore, we tested the performance after three different modifications, each of which challenges an assumption we make in building this pipeline. Testing these changes alone still leaves open the possibility that a combination of them, and possibly additional ones, would lead to stronger classification performance. For computational feasibility, however, we evaluate the effect of each change alone relative to our baseline.

The first modification we call “Target-down”, which only accepts gene-disruption samples if the supposed target gene had decreased expression compared to the control samples (drug treatment data are kept as they are). This accounts for the possibility that the tool’s performance is hampered by studies where either the methodology, the labeling, or the curation was improper. The second modification we call “ARCHS4-only”, which only uses samples from ARCHS4 (the largest of the three read count datasets), rather than incorporating Recount3 and DEE2. Ideally, all read counts should be calculated using the same alignment tool, so limiting to one challenges our assumption that it is safe to conflate signatures calculated on different platforms. The third modification, which we call “Mouse”, uses mouse data instead of human. KO/KD data are far more abundant in mouse tissue than human, so the former may offer more robust predictions.

### Indirect predictions of regulatory relationships are derived from signature-matches

A major contribution offered by PARMESAN is its ability to predict undiscovered regulatory relationships by linking the known ones together. We have demonstrated its ability to expand our repertoire beyond what was explicitly written in the literature(12), and the same principle may apply to SNACKKSS and bolster its predictive ability.

Notably, gene coexpression networks are a common means of identifying pairs of genes that may have similar or related functionality(16, 18). Unlike signature-matching, they do not establish temporality—when two genes have increased expression, we do not know whether one increase caused the other. However, it offers a far larger repertoire of potential relations than the signature-matching approach does. ARCHS4 provides a readily accessible table of Pearson correlations between the expression of any two genes (covering 29,082 human and 24,530 mouse genes), across all of the human and mouse samples that they curated. The correlations are a powerful predictor of supportive gene-gene relations (see Results), so we investigated whether they could improve the literature- and RNA-based relationship predictors. We first linked PARMESAN’s gene-gene relation consensuses to either its own consensuses (as PARMESAN normally does to make indirect predictions), or to said correlations. Our linking formula simply treats ARCHS4’s correlation coefficients as gene-gene relationship consensus scores, which we feed into the I_CA_ formula as described in Deisseroth et al(12).

Similarly, we test the performance of linking SNACKKSS’ DF1 scores to ARCHS4’s correlations, treating the former the same way we treat PARMESAN’s consensus scores. We evaluate the performance using the default setup for SNACKKSS, as well as with the three aforementioned permutations, Target-down, ARCHS4-only, and Mouse.

### The Connectivity Map is fed into the same relationship prediction pipeline

As we did with SNACKKSS, we tested the ability of the Connectivity Map to correctly identify regulatory relationships. Specific details on our processing of Connectivity Map data are provided in Supplemental methods. In addition to testing a default prediction pipeline, we introduce four modifications that challenge unique assumptions we make regarding our approach. The first, “Inferred”, uses all of the quantified gene expression levels, instead of just the landmarks. The second, “shRNA only”, excludes CRISPR KO data when calculating gene-disruption signatures.

The third we call “MCF7”, which only uses samples in the MCF7 cell line, and tests the effect of taking the tissue type into consideration. Although SNACKKSS is implicitly trained to select control samples that are of the same tissue type as a perturbed sample, it does not consider tissue type when calculating consensus signatures—which, one could argue, might hinder its performance. Quantifying the comparability of samples from different studies would require not only a strict encoding of plain-text tissue descriptions in GEO, but also a ground truth for defining two samples as comparable to each other. CMap, however, does have a strict encoding of the tissue used. Hence, we tested the performance from using only samples from their most-tested cell line, MCF7.

The last modification we call “OE-corrected”. Correlations between the signatures of two perturbations can reflect their relationship, but it can also be due to a cellular stress response or off-target effects. Overexpression studies would theoretically be a promising solution to this conundrum: DEGs that change in the same direction in response to a KO/KD or OE of Gene A are likely unrelated to the altered level of A, but if the KD and OE change a gene’s level in opposite directions, that DEG is far more likely to truly be responding to A’s level, thus giving us a cleaner and more faithful signature(44). Unfortunately, per both our curation and that of CREEDS, OE studies are less common than KO and KD, but with CMap’s repertoire of OE data for 3,542 target genes (2,753 of which also have either KO or KD data), we may be able to test this hypothesis. Hence, this final modification only uses target genes that have both OE and KO/KD data, takes the average magnitude between the two, and excludes any DEGs that changed in the same direction.

### Other predictive tools were evaluated

Ultimately, we have the following candidate predictive tools:

“SNACKKSS”: Using the signature-matches from SNACKKSS (default setup) to predict regulatory relationships. Positive DF1 scores are interpreted as supportive gene-gene and inhibitory drug-gene relations, and negative scores are interpreted as inhibitory gene-gene and supportive drug-gene relations.

“CMap”: Using the signature-matches from the Connectivity Map (default setup) to predict regulatory relationships, as we do with SNACKKSS.

“PARMESAN consensus”: Using the consensus scores from PARMESAN, as we have done previously(12).

“PARMESAN hypotheses”: Using the indirect relationship predictions from PARMESAN, as we have done previously(12).

“PubTator3 consensus” Feeding PubTator3’s extracted regulatory relationships into PARMESAN’s consensus formula.

“PubTator3 hypotheses”: Feeding PubTator3’s extracted regulatory relationships into PARMESAN’s consensus formula, and feeding those consensuses into the indirect prediction formula.

“Human A4C”: Using the Pearson correlations from ARCHS4’s human gene coexpression matrix to predict gene-gene relations. Positive and negative correlation coefficients are interpreted as predictions of supportive and inhibitory gene-gene relations, respectively.

“Mouse A4C”: Same as “Human A4C”, except using ARCHS4’s mouse gene coexpression matrix instead.

“SA4”: Linking the signature-matches from SNACKKSS to A4C, as described above.

“CMA4”: Linking the signature-matches from CMap to A4C, analogously to SA4.

“PA4”: Linking PARMESAN’s consensuses to A4C, instead of to its own gene-gene relationship consensuses.

“P3A4”: Linking the consensuses from PubTator3 to A4C, similarly to PA4.

### Redundancy of SNACKKSS’ predictions is assessed

SNACKKSS, whether alone or when linked to ARCHS4’s correlations, is outperformed by another tool for every prediction task (see Results). Its poorer coverage, however, does not necessarily mean that it should not be used. It draws relationships from a data source nigh untouched by PARMESAN and PubTator3, and can identify relations that would have been missed when using the literature alone. Nevertheless, incorporating a noisy predictive tool that sometimes looks accurate through pure chance can ultimately hinder prioritization of candidate drugs (see Table 2 for examples). Therefore, our final assessment of this tool’s utility is a simulation of using an assortment of other predictors, or using SA4 alongside them to prioritize candidates.

Our setup is akin to LOOCV: For a given drug A known to modulate a given protein B, we exclude any relations involving A or B from DGIdb, resulting in the database DGIdb_-AB_. For each predictor (PubTator3, P3A4, PARMESAN, PA4, and SA4), we measure the accuracy of the scores above any given threshold in predicting the directionality of the relations in DGIdb_-AB_, thereby mapping its directionality scores to accuracy estimates â. Then, when predicting the relation between A and B, each of the predictors will provide a putative directionality and an â value. The directionality we posit is that of whichever predictor gave the highest â, and we score the prediction with that â value. The resulting accuracy, a, is the percent of left-out A-to-B relations that were correctly predicted when we only accepted predictions with â values above a given threshold.

This simulation represents a user’s approach to using multiple tools to predict a relation that has not been discovered: if none of the tools make a confident prediction, the candidate drug will have low priority, and if SA4 is redundant with the other predictors, then it will not confidently predict any “undiscovered” relationships that the other tools could not predict more confidently. This simulation also accounts for the imperfection of our reference databases: SA4 might yield overly confident predictions that appeared strong amongst the relations covered by DGIdb, but ultimately assign the wrong directionality to relations that PubTator3 and P3A4 were not confident about, thus worsening the overall performance. Again, we compare the coverage with versus without SA4 using a binomial test for the proportion of accuracy levels we were able to outperform by adding SA4 (with an expected rate of 0.5)—but we reiterate that this test statistic has few practical implications, as the best model to use can depend on the accuracy one is willing to accept.

### Statistical analyses

Statistical methods are described in their appropriate sections. All p-values are two-sided. When comparing the accuracy of our BERT classifiers to one another, we did not use any statistical tests, because it does not matter to us whether the differences in performance could occur due to random chance. One could potentially test for a significantly improved performance using two-proportion Z-tests for precision and recall, but our goal is to decide which model to use, and significance does not affect this decision. While DistilBERT is faster than the other two models, the tasks in this pipeline are feasible for all three, so we would use BioBERT or BioMedBERT if it performed even slightly better than DistilBERT.

95% confidence intervals around percentages are calculated using a binomial distribution via Microsoft Excel’s BINOM.INV function. For smoothed F1 scores, 95% confidence intervals are calculated by measuring the binomial 95% confidence interval (same formula) around the smoothed precision and recall.

We display numerators and denominators without modification, but when calculating proportions, we increment the number of undesirable results by one. This is similar to Bayes-Laplace Smoothing (BLS), except that BLS brings all proportions closer to 50%, which penalizes small sample sizes for proportions above 50% while rewarding them if they are below 50%. This more-stringent version of BLS, by not incrementing the number of successes, penalizes small sample sizes regardless of the proportion.

All p-values given are unadjusted unless stated otherwise. We used Bonferroni correction (i.e. multiply all p-values by the number of hypotheses tested) for all multiple-hypothesis adjustments, and would do so if and only if, among the multiple hypotheses tested, we would consider at least one of them succeeding to be an overall success. For example, when we display the performance of SNACKKSS at different DEG thresholds, we do not run any correction, because we would only consider the predictor successful if it performed well under LOOCV. However, when measuring the redundancy of SA4 with other predictors, we are testing four different hypotheses (whether it improves our predictive ability for positive and negative gene-gene and drug-gene relations), and would endorse this predictor if any of them succeeded; therefore, we must adjust the p-values by multiplying them by four.

## Results:

### BERT-based metadata classification was not stable across machines

**Figure 1:**
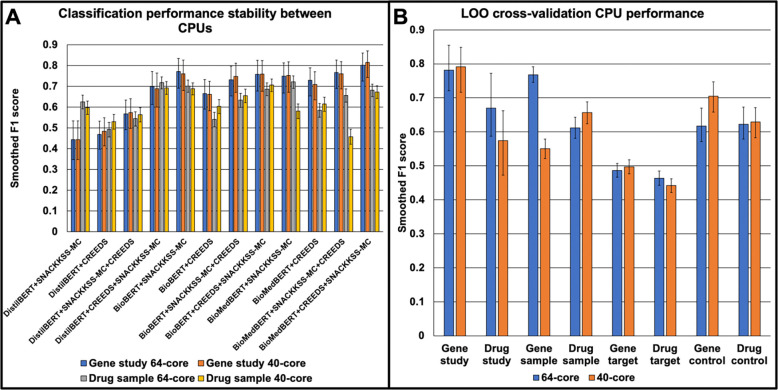
BERT models can perform differently on different machines. (**A**) Through 4-fold cross-validation, we trained DistilBERT, BioBERT, and BioMedBERT on either SNACKKSS-MC or the CREEDS dataset, or both (in both orders) on a 64-core and a 40-core CPU, for gene-disruption study and drug-treatment sample classification, and tested their performance on SNACKKSS-MC. We measure the performance as a smoothed F1 score. (**B**) We have each machine identify and use its optimal model for each task through leave-one-out cross-validation, and the smoothed F1 scores can differ between them, from less than 1% to more than 20%. The error bars are calculated by measuring the binomial 95% confidence interval around a predictor’s precision and recall values, and calculating the F1 scores resulting from the minimum and maximum values.

We evaluated the performance of each BERT model trained on each manually curated corpus, for each of the eight classification tasks: study, sample, target, and control classification for gene-disruption and drug-treatment experiments. Despite containerization of our pipeline, locked random seeds, and parameters that we verified to result in replicable training and testing when run on one machine; we observed that the BERT models produced different output on different machines (Supplemental Table 4 displays the accuracy metrics from running the pipeline on a 40- and 64-core CPU), and sometimes even reached different conclusions on which model performs the best. Figure 1A shows examples of stable (gene-disruption study) and unstable (drug-treatment sample) classification performance. For the former, the models’ smoothed F1 scores were always less than 3% different between CPUs, while for the latter, they could be as much as 19% different. However, the accuracies of the respective top-performing models from each CPU were always less than 2% apart. This led us to ask whether, if we let each CPU select and run its top-performing model, they ultimately converge on similar accuracy. To test this, we ran a form of leave-one-out cross-validation (LOOCV), where having trained each model on the other three 4-fold cross-validation splits and tested it on the fourth, we use all of the GEO series from the testing split except one to determine which model is the most accurate, and use that model’s predictions for that left-out series. Figure 1B shows the resulting F1 scores achieved by the two CPUs, and the differences between them could range from 0.7% to 22%. This instability is important to be aware of, but it does not preclude the use of BERT models for automated curation, because the reasoning of deep-learning models is difficult to trace regardless, and since the classifications are saved, any downstream prediction errors can be traced back to the BERT curation if need be. Hence, there is still merit in testing the ability of the saved output to guide relationship predictions.

Using the 40-core CPU, our final gene-disruption study curation pipeline used BioMedBERT trained on SNACKKSS-MC alone for gene sample and drug target classification, BioBERT trained on CREEDS’ dataset followed by SNACKKSS-MC for drug samples, and BioMedBERT trained on CREEDS’ followed by SNACKKSS-MC for the other five tasks. To give one successful example, this pipeline accurately annotated GSE160415 (per the 4-fold cross-validation training BioMedBERT on our dataset alone on the 40-core CPU). This study tested knockout of *Mark3* in mouse osteoblasts. The gene- and drug-study classifiers marked this study as positive and negative, respectively. The sample classifier identified GSM4873066, GSM4873067, and GSM4873068 (and no other samples) as receiving a gene KO/KD; and the target classifier exclusively procured “Mark3” instances from their descriptions. For each KO sample, the control classifier marked all of the wild-type samples as controls—although this particular study had no candidate controls that were not in fact controls. Meanwhile, this pipeline mislabeled the study GSE224420 (testing *ROR2* knockdown in a cancer cell line(45)) as non-gene-disruption. These examples are meant to illustrate the type of information extracted by SNACKKSS. Indeed, we cannot trace the reasoning of these labels, but we can trace downstream errors back to the labels themselves.

### Gene expression changes are largely concordant with SNACKKSS’ study annotations

Having established pipelines for curating gene-disruption and drug studies, we next measured the value of interpreting these automatically curated studies. We ran our two pipelines on all of the qualified studies, and identified which samples had read counts in ARCHS4 (from which we extracted 79,835 human samples, 20,234 of which received a gene- and 19,968 a drug-perturbation; and 114,507 mouse samples, 43,630 gene- and 12,980 drug-perturbed), Recount3 (26,012 human, 6,083 gene- and 6,974 drug-perturbed; and 42,703 mouse, 16,730 gene- and 3,567 drug-perturbed), and DEE2 (32,303 human, 8,125 gene- and 8,046 drug-perturbed; and 40,951 mouse, 16,229 gene- and 4,045 drug-perturbed). We found that across all read count datasets, the supposedly perturbed target genes predominantly decreased in expression in both humans and mice (Numbers of samples with increased and decreased target gene expression are plotted in Supplemental figure 2 and listed in Supplemental table 5). The binomial p-values (Bonferroni-corrected for six hypotheses) were all undetectably low for human ARCHS4 (81.0%, 15,413/19,025, 95% CI 80.5–081.6%), mouse ARCHS4 (73.6%, 29327/39858, 95% CI 73.1–74.0%), human Recount3 (83.3%, 4815/5779, 95% CI 82.3–84.3%), mouse Recount3 (72.5%, 11005/15184, 95% CI 71.8–73.2%), human DEE2 (78.7%, 11362/14442, 95% CI 78.0–79.3%), and mouse DEE2 (71.9%, 16304/22666, 95% CI 71.3–72.5%). Granted, the expression-increases ranged considerably higher than the decreases, likely because expression levels can go to infinity, but not below zero. Ultimately, disrupted genes were far more likely to show decreased expression than increased, which suggests that they and their controls were effectively identified.

### Matching disruption signatures yields weak predictions of gene modulators

**Figure 2:**
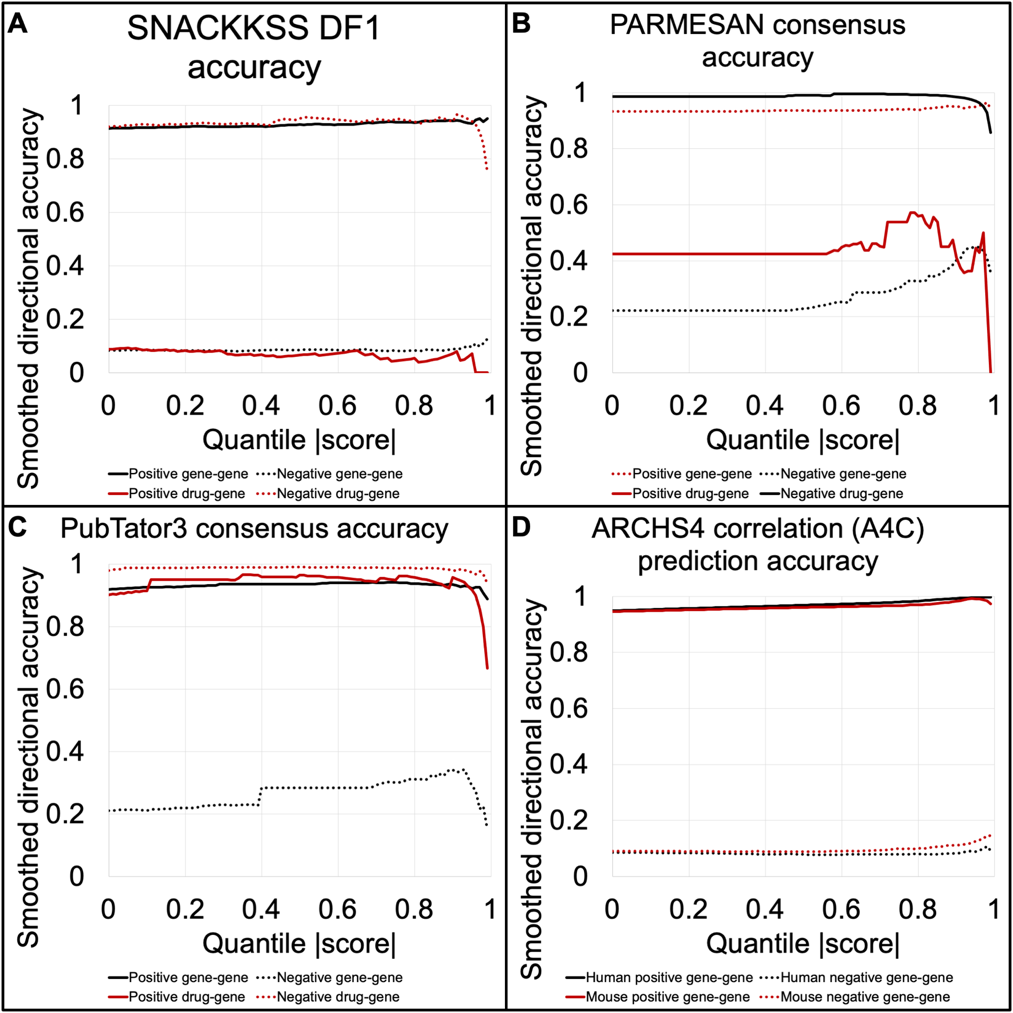
Whole-signature matching is a weak predictor of regulatory relationships We measure the likelihood of getting the correct directionality (up- versus down-regulation) among the relationships posited by SNACKKSS (A), PARMESAN’s consensuses (B), PubTator3’s consensuses (C), or ARCHS4’s expression correlations (D). The X axis is the minimum score quantile above which we accept putative relations—for example, at 0.9, we accept only the top-10%-scoring candidates. The manually curated databases (MCDBs) we use as a gold standard are Reactome (gene-gene) and DGIdb (drug-gene relations). If a prediction tool is effectively prioritizing correct relations over incorrect ones, its accuracy should increase with higher scores (and, by extension, score quantiles).

We tested the high-speed signature-matching algorithm, Differential F1 (DF1), for its ability to predict the directionality (up- or down-regulation) of the manually curated regulatory relationships provided by Reactome (functional interactions where one protein supports or opposes another(36)) and DGIdb (The Drug-Gene Interaction Database, indicating molecules that support or oppose specific gene products(35). Accuracy across score thresholds is plotted in Figure 2A, with log-rank test statistics for all signature-matching-based predictors provided in Supplemental table 6 and raw correct/incorrect counts provided in Supplemental table 7. To give one example of a successful prediction, DGIdb asserts that gefitinib, an epidermal growth factor receptor inhibitor(46) (Also known as ZD1839 or “Iressa”, PubChem SID 532631), opposes Human Epidermal Growth Factor Receptor 2 (HER2, also known as ERBB2, Entrez ID 2064), and there are multiple indirect mechanisms through which it is theorized to do so(47). Using the relationship predictions involving neither gefitinib nor any of its targets, 0.3 was the best DEG z-score threshold for prioritizing correct inhibitory relations over incorrect ones, and 0.9 was the best for prioritizing supportive relations. Using 0.3 as the threshold, across the gefitinib treatment and *ERBB2* disruption studies (in human tissue), there were 9,991 DEGs that went in the same direction (STP), 9,797 in the gefitinib but not the *ERBB2* disruption signature (FP), 20,159 in the *ERBB2* disruption but not the gefitinib signature (FN), and 4,283 shared DEGs that went in opposite directions (OTP). With the above formulae, SP = 0.415, SR = 0.331, SF1 = 0.236, OP = 0.178, OR = 0.175, OF1 = 0.0691, and DF1 = 0.0428, suggesting that gefitinib yields a similar effect on the transcriptome to disruption of *ERBB2*, and likely opposes it. Using 0.9 as the threshold, STP = 405, OTP = 222, FP = 2,625, FN = 21,424, SP = 0.125, SR = 0.0186, SF1 = 0.00606, OP = 0.0682, OR = 0.0103, OF1 = 0.00195, and DF1 = 3.36×10^−5^ —a weaker match, but in agreement with the negative-optimized match, hence we keep our prediction. This is only one example of matching signatures coinciding with the relationship between those two entities, and we must evaluate whether the principle holds true across the regulatory relationships in Reactome and DGIdb.

Before taking directionality into consideration, the gene-gene relations seemed to be prioritized well (all log-rank test statistics > 6), while the drug-predictions were not (all < 1.7, Supplemental table 6, Figure 2A). However, we observed a shared bias between SNACKKSS and Reactome. Positive correlations between perturbations (which suggest positive gene-gene and negative drug-gene relations) are more common than negative ones, with stronger correlations being more likely to be positive (limiting to the signature-matches involving known modulators in Reactome and DGIdb, log-rank p < 10^−22^ for both gene-gene and drug-gene matches, at all DEG thresholds except 0.9 for drug-gene relations, where p = 0.141); Reactome contains 100,724/106,656 (94.4%, 95% CI 94.3–94.6%) positive relations; and DGIdb contains 18,616/25,313 (73.5%, 95% CI 73.0–74.1%) negative relations. To address this bias, we analyzed positive correlations separately from negative ones. If shared directional bias is the sole contributor to accuracy improvements, then when we guarantee a fixed directionality of our predictions, the relations to which the MCDB ascribed a positive and negative directionality should decline at the same rate as we increase the score threshold. Therefore, this separation can assess whether an increase in the magnitude of the score truly improves accuracy.

Unsurprisingly, the prioritization was notably weaker after this separation (log-rank p = 2.07×10^−6^ for positive gene-gene, 0.982 favoring the wrong direction for negative gene-gene, 0.251 favoring the wrong direction for positive drug-gene, and 0.205 for negative drug-gene relations. After Bonferroni correction for 72 hypotheses, these p-values are 1.49×10^−4^, 1, 1, and 1, respectively). For PARMESAN, this was not as severe of an issue (Figure 2B, log-rank test results provided in Table 1). However, this caveat seen in SNACKKSS informed us that, even for PARMESAN, we must consider the two directions separately when we estimate accuracy, as scores of −1 and +1 are not necessarily equally reliable.

Existing resources can provide analogous regulatory relationship predictions that can be tested for accuracy in the same way. PubTator3’s extracted gene-gene and drug-gene relations can also be fed into PARMESAN’s consensus formula, and they demonstrate strong accuracy (Figure 2C). Furthermore, ARCHS4’s gene coexpression matrix can be used for the same purpose, where a stronger correlation coefficient is interpreted as a more-confident prediction. From comparing ARCHS4’s gene expression correlations in human tissue to the known regulatory relationships in Reactome, strong positive correlations were a decisive indicator of positive gene-gene relations, with substantially better coverage than PARMESAN (Figure 2D). The negative relations, however, were not as robust: there was no significant prioritization from human data, and although there was in mice, the accuracy in identifying them never reached 15%, which we do not expect most users to find acceptable—thus, one might gravitate toward PARMESAN for this purpose instead. Intuitively, each prediction task is performed best by a different algorithm.

### Compared to human data, mouse data yield better identification of gene-gene relations

To assess for flaws in our approach to signature-based relationship predictions, we tested the performance of SNACKKSS after implementing three methodological permutations: “Target-down”, which only accepts gene-disruption samples if the supposedly disrupted gene had decreased expression compared to the controls; “ARCHS4-only”, which only uses pre-computed read counts from ARCHS4; and “Mouse”, which uses mouse data instead of human. We plot the performance of SNACKKSS after each permutation (Target-down, ARCHS4-only, and Mouse) in Supplemental figure 3, with the log-rank test statistics for prioritization of correct over incorrect in Supplemental table 6 and correct/incorrect counts in Supplemental table 7. Because we are measuring whether any permutation can salvage the performance of our predictor, we run Bonferroni correction for 72 hypotheses. In this regard, none of the permutations led to strong prioritization of drug-gene relations (the best result was from the Target-down approach, p_adj_ = 0.226 for inhibitory and 1 for supportive relations), nor inhibitory gene-gene relations (with the best performance from Target-down, p_adj_=1).

### ARCHS4’s expression correlations can bolster other predictive tools

**Figure 3:**
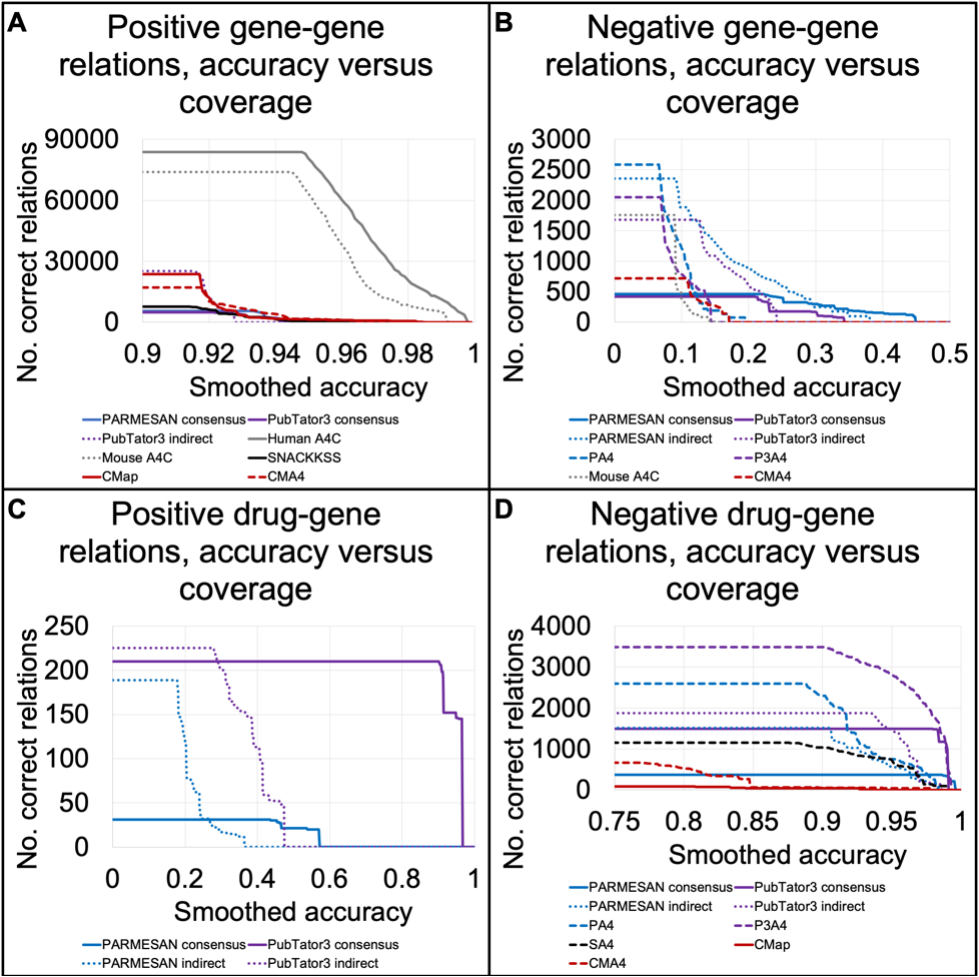
Signature matches synergize with coexpression data and contribute to negative drug-gene relationship identification We display the performance of different indirect tools in identifying positive gene-gene (A), negative gene-gene (B), positive drug-gene (C), and negative drug-gene (D) relations. Specifically, we plot the accuracy that each predictor achieved (X axis) against the number of correctly identified manually-curated-database (MCDB) relations above the lowest score that yielded that accuracy (Y axis). This is not a precision-recall curve, but it follows the same principle that a strong predictor has at least one point with a large X and Y value. The predictors are PARMESAN’s consensuses, indirect hypotheses, and ARCHS4-linked predictions (“PA4”), PubTator3’s consensuses, indirect hypotheses, and ARCHS4-linked predictions (“P3A4”), gene expression correlations from ARCHS4’s human and mouse datasets (“Human A4C” and “Mouse A4C”, respectively), signature-matches from SNACKKSS or Connectivity Map (“CMap”), and ARCHS4’s correlations linked to signature matches from SNACKKSS (“SA4”) or Connectivity Map (“CMA4”). We then evaluate the performance of SA4 (SNACKKSS/ARCHS4 hybrid) (C). We only plot a predictor if it prioritizes correct predictions over incorrect ones, with an unadjusted log-rank p < 0.05.

Due to the strong performance observed from using ARCHS4’s co-expression matrix to predict supportive gene-gene relations, we asked whether linking them to literature-based consensuses offered augmented coverage, and if so, whether that came at a cost to accuracy. The correct/incorrect counts across score quantiles for PARMESAN- and PubTator3-derived indirect predictions, and for those derived from linking PARMESAN or PubTator3 to ARCHS4’s expression correlations (PA4 and P3A4, respectively) are in Supplemental table 8, with log-rank statistics provided in Table 1. When measuring the number of gene-gene and drug-gene relations each predictor correctly identified above the smallest score achieving each accuracy level (plotted in Figure 3), P3A4 offered record-breaking coverage across accuracy levels for finding inhibitory drugs.

When we linked SNACKKSS’ gene-gene DF1 scores to ARCHS4’s correlations (“SA4”, with log-rank prioritization statistics in Supplemental table 6, raw correct/incorrect counts across score thresholds in Supplemental table 9, and number of correct predictions across accuracy levels in Figure 3), we only observed strong prioritization of inhibitory drugs (log-rank p = 1.23×10^−29^, p_adj_ = 8.85×10^−28^). Among the drug-gene relations verified by DGIdb, the highest-scoring inhibitory relationship from SA4 was from indisulam (PCID 12015040) to GAPDH (Entrez ID 2597), with a z-threshold set as 0.2 through LOOCV, resulting in a score of 3.58. DGIdb agreed that this relationship was inhibitory, citing Guide to Pharmacology(48). The single strongest contributor to this prediction (with the other contributors provided in Supplemental table 10) involved similar signatures of indisulam treatment and disruption of *PPM1G* (Entrez ID 5496, DF1 = 0.183), a gene whose expression has a correlation coefficient of 0.503 with that of *GAPDH*. Note that this one correct prediction is only meant to serve as an example, not as evidence for or against the performance of this method. The global comparison to DGIdb, however, does suggest effective prioritization of inhibitory substances.

As we did with direct signature-matching, we measured the effect of limiting to samples with down-regulated targets, limiting to ARCHS4, and using mouse data (full performance statistics are in Supplemental table 6, correct/incorrect prediction counts across score thresholds are in Supplemental table 9, and accuracy is plotted against number of correct predictions in Supplemental figure 3). Mouse data improved the gene-gene relationship predictions (for positives, p = 2.33×10^−5^, p_adj_=0.00168; for negatives, p = 0.000886, p_adj_=0.0638), nothing salvaged the positive drug-gene relations, and nothing sharply improved the negative drug-gene relations. While one would normally be inclined, in light of these results, to use the permutations that improved SNACKKSS’ performance, doing so may compromise our benchmarking, as these changes would constitute hyperparameter-tuning, and thus run the risk of overfitting to Reactome and DGIdb. Future work may include using cross-validation to select and evaluate the optimal method, which would require additional streamlining due to the runtime-multiplicity of testing different combinations of features. For this study, we adhere to the default regardless of the outcomes observed, and compare permuted pipelines purely for investigational purposes. Beyond the use of mouse data, this test did not identify any obvious ways to improve SNACKKSS’ performance.

### Connectivity Map data function similarly to SNACKKSS, but involve different perturbagens

SNACKKSS uses public RNA-Seq data to predict regulatory relationships, but it is not the first systematic, large-scale application of the signature reversion paradigm. The Connectivity Map(21) (CMap) is a massive series of microarrays measuring the levels of 978 genes in multiple human cell lines perturbed with tens of thousands of compounds, shRNAs, CRISPR knockouts, and overexpression vectors. Despite the limited breadth of the microarray, the database provides all of the features that we have been trying to automatically annotate, in a machine-readable format, as well as the cell line, which SNACKKSS does not definitively account for. Because their database is interchangeable with the signature data that SNACKKSS uses, we cannot say that SNACKKSS makes a novel contribution until we know whether the same contribution could be made when matching signatures from CMap instead.

CMap imputes the expression of over 11,000 genes, and its definition of “best inferred” is that the predicted expression level has a statistically significant correlation with the actual level. However, a statistically significant correlation is not the same as a reliable one. To assess the usability of these inferences, we first measured the differential expression of the targeted genes in the shRNA, CRISPR KO, and overexpression samples, in comparison to the control samples (control vector, control vector, and DMSO, respectively) from the same cell line. Bonferroni correction accounts for nine hypotheses. For the landmark genes, there was decreased expression of 84.7% (10,537/12,443, p undetectably low, p_adj_ undetectably low, 95% CI 84.0–85.3%) of the CRISPR targets, 84.6% (47,957/56,665, p undetectably low, p_adj_ undetectably low, 95% CI 84.3–84.9%) of the shRNA targets, and 33.3% (2,782/8,340, p = 1.11×10^−206^, p_adj_ = 9.98×10^−206^, 95% CI 32.4–34.4%) of the overexpression targets (Supplemental figure 4A, Supplemental table 5). For the best inferred genes, these percentages were 54.2% (33,349/61,519, p undetectably low, p_adj_ undetectably low, 95% CI 53.8–54.6%), 48.2% (43,906/90,999, p = 4.44×10^−26^, p_adj_ = 4.00×10^−25^, 95% CI 47.9–48.6%), and 46.2% (7,743/16,746, p = 2.17×10^−22^, p_adj_ = 1.95×10^−21^, 95% CI 45.4–47.0%), respectively (Supplemental figure 4B), and for the inferred genes, they were 51.0% (6,660/13,058, p = 0.0214, p_adj_ = 0.192, 95% CI 50.1–51.9%), 46.7% (3,146/6,731, p = 9.27×10^−08^, p_adj_ = 8.34×10^−07^, 95% CI 45.5–47.9%), and 44.4% (763/1,718, p = 3.98×10^−06^, p_adj_ = 3.58×10^−05^, 95% CI 42.1–46.7%), respectively (Supplemental figure 4C). The strong concordance between disruption of landmark genes and their decreased expression suggests that the direct measurements were accurate and the gene-perturbations were usually effective. Given this, the relative lack of concordance seen from imputed gene expression levels suggests, as expected, that they are not nearly as reliable as the direct measurements.

We next determined whether matching signatures from CMap led to accurate predictions of regulatory relationships. Using DF1, we compared KO/KD signature matches to Reactome relations, and compound-to-KO/KD matches to DGIdb relations (log-rank prioritization statistics are in Supplemental table 6, correct/incorrect counts are in Supplemental table 7, and accuracy is plotted against number of correct predictions in Figure 3). Upon matching signatures, we observed decent prioritization of positive gene-gene relations (log-rank p = 9.11×10^−6^ for positive gene-gene, 0.347 favoring the wrong direction for negative gene-gene, 0.000255 favoring the wrong direction for positive drug-gene, and 0.000973 for negative drug-gene relations. p_adj_ = 6.56×10^−4^, 1, 0.0184, and 0.0701, respectively).

As we did with the other predictors, we determined whether the DF1-based predictions from CMap could be bolstered when linked to the expression correlations from ARCHS4 (“CMA4”, log-rank prioritization statistics are in Supplemental table 6, correct/incorrect counts are in Supplemental table 9, and accuracy is plotted against number of correct predictions in Figure 3). This linking led to improved prioritization of negative gene-gene relations (Log-rank p = 2.16×10^−8^ for positives and 2.21×10^−6^ for negatives, p_adj_ = 1.55×10^−6^ and 1.59×10^−4^, respectively).

Among the default and permuted CMap-derived predictors, four and three effectively prioritized positive and negative gene-gene relations, respectively; and one and seven effectively prioritized positive and negative drug-gene relations, respectively. Correct and incorrect counts across score thresholds are provided in Supplemental table 9, log-rank statistics are in Supplemental table 6, and accuracy is plotted against number of correct predictions in Supplemental figure 5). This demonstrated some merit to limiting to a specific tissue type, and—to our surprise—using inferred expression levels.

### Inhibitory drug predictions are non-redundant with other tools

SA4 alone is not the strongest predictor in any regard. Since it uses a unique source of information, however, it might find regulators that the other tools never would have considered, and could thus be a beneficial addition to one’s repertoire. Nonetheless, including a new predictor in a decision process can be helpful or harmful (Table 2). To test what SA4 adds beyond what other, more-powerful predictive tools do, we use LOOCV to estimate the accuracy of each predictor, and use the most-confident prediction for the left-out relation.

**Figure 4:**
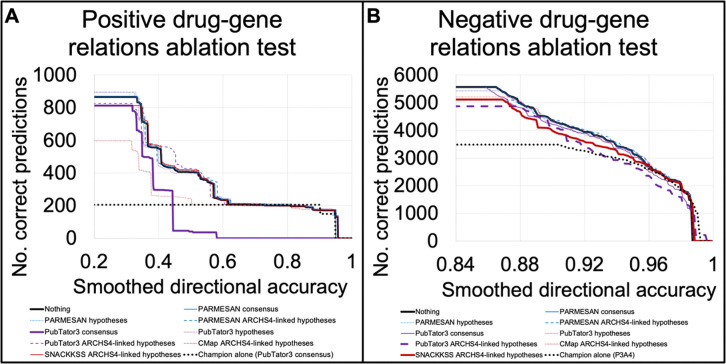
Contribution of each predictor to drug prediction Through leave-one-out cross-validation (LOOCV), we evaluated the use of all predictors tested in this study to identify previously-unseen drug-gene relations. Here, we test the contribution of each predictor to such an effort by removing it from the repertoire and measuring how the performance changes. We plot the accuracy achieved against the number of correctly identified positive (A) and negative (B) drug-gene relations above the lowest confidence threshold yielding that accuracy. Whether a predictor is helpful usually depends on the accuracy one is willing to accept. Hence, depending on where a user sets the cutoff, some predictors—even if they do well on their own—will do more harm than good when used alongside more powerful tools. SA4 yields improved coverage in finding negative drug-gene relations, and for drug prediction overall, the ensemble of predictors yielded better coverage than using the reigning champion alone.

We determined the number of correctly identified regulatory relationships across accuracy levels when using all predictors (and believing the most confident one), and using all of them except one. To provide a simplistic account of the effects of adding each predictor, Table 3 indicates whether doing so increased the maximum accuracy, and whether it increased the overall number of predictions made. This is by no means a comprehensive description of the benefits of each predictor, and benefiting one facet would often come at a cost to the other. Users should ultimately decide which set of models to use based on their own preferred balance of accuracy against coverage, and we display the coverage at each accuracy level upon removing each predictor in Supplemental table 11, Figure 4 (drug-gene relations), and Supplemental figure 6 (gene-gene relations), and the fraction of accuracy thresholds for which each predictor improved coverage in Supplemental table 12, with 95% confidence intervals around the percentage, binomial test statistics, p-values with and without Bonferroni correction for 40 hypotheses. Individual predictors’ accuracy/coverage distributions in the LOOCV test are provided in Supplemental table 13. We did not test the performance of removing multiple predictors, because the process of finding the top-performing combination would transcend the LOOCV and overfit to the MCDBs, and the resulting accuracy metrics would be unreliable. Our goal with this test is to showcase the tradeoff (or the strict benefit or burden) of each predictor. And indeed, each one usually did introduce such a tradeoff—but we again see A4C’s strong contribution to the identification of supportive gene-gene relations, and P3A4’s benefit in inhibitory drug prediction.

Adding SA4 was highly beneficial for negative drug-gene relations (outperforming 394/413 accuracy levels, binomial p and p_adj_ undetectably low), detrimental for positive ones (outperforming 10/112 accuracy levels), and detrimental for both positive and negative gene-gene relations (outperforming 207/2563 accuracy levels for positive relations, and 1/15 for negatives). Furthermore, the aggregation of these predictors led to vastly improved coverage in identifying drug-gene relations, compared to using the best predictors alone (PubTator3’s consensuses for supportive relations, and P3A4 for inhibitory ones), while for supportive gene-gene relations, it was comparable to using just ARCHS4’s correlations.

Unexpectedly, for negative gene-gene and positive drug-gene relations, removing datasets from this LOOCV evaluation sometimes led to more predictions. Indeed, our system is designed to believe whichever model is the most confident in its selected direction, and with all models inherently being more confident in one direction than the other, they can serve as a filter for candidate relations in the rarer direction. In other words, all models more confidently predict positive than negative gene-gene relations, so in order for this LOOCV system to predict a negative relation, none of the models must confidently say it is positive. For inhibitory gene-gene relations, this seemed to detract from the effort, as using PARMESAN’s indirect hypotheses alone yielded overall stronger performance than the ensemble. This phenomenon could be an indication to train a sophisticated machine-learning classifier on the scores assigned by different models (as others have done for their ensemble predictors(23)), and future work may include exploring some of the infinitely many possible implementations.

### Most predictive tools introduce an accuracy-coverage tradeoff, with SA4 distinctly benefitting inhibitory drug prediction

Since we know that SA4 makes a unique contribution to our overall predictive effort, we sought to answer the question, for a given gene product that one is studying, how much have we improved the likelihood that one will be able to find a promising inhibitor of it? We plot the number of genes for which we were able to correctly identify an inhibitor across accepted accuracy levels, before and after adding SA4 to our repertoire (Supplemental table 14 and Supplemental figure 7). For example, if one desires 95% accuracy, SA4 allows one to target 40 more genes than one could without it, underscoring its benefit to drug-repurposing efforts.

## Discussion:

There are tens of thousands of rare genetic disorders that still lack targeted therapies, but are not studied widely enough that we can identify promising candidate regulators. The millions of publicly available RNA-Seq samples offer tremendous promise in guiding drug discovery for these conditions, and many researchers have developed innovative ways to make them findable, accessible, interoperable, and reusable(49); but there has been one missing step required for feeding them into fully automated drug prediction: a machine-readable registry of gene-disruption and drug-treatment studies. SNACKKSS fulfills this role and carries it directly into the effort to identify molecules that can up- or down-regulate specific gene products. While it is not the best solitary predictor in any regard, we demonstrate that adding it to our repertoire substantially improves our ability to identify inhibitory drugs, even in the wake of other predictive tools.

Along with our findings, we offer four key resources to the public, which will be available at snackkss.nrihub.org upon submission for peer review. The first is a database of automatically curated RNA-Seq experiments from GEO testing gene disruptions and drug treatments, detailing the control samples that correspond to each perturbed one, as well as what perturbation was administered. As with any automatically curated dataset, the accuracy is not perfect, and these labels should not be taken as a ground truth. Nonetheless, they will likely be an asset to users who want to systematically gather previous experiments testing a specific drug or gene disruption.

The second is the consensus signatures of each gene disruption and drug treatment (searchable by both perturbagen and differentially expressed gene), and the third is a database of predicted regulatory relationships, automatically constructed from the SNACKKSS and SA4 approaches. However, we provide the disclaimer that we only expect the inhibitory drug predictions from SA4 to offer a valuable contribution to the identification of novel protein regulators. The fourth is the ensemble of the different predictors we tested (for download only, and only for drug-gene relations), which displays each tool’s prediction and confidence, and which we expect to be a particularly strong advance in drug prioritization.

While SNACKKSS offers a systematic implementation of the signature reversion paradigm, it is not a definitive evaluation of the principle itself. There are several major limitations in this study—first and foremost, the signature that a treatment is reversing has to be part of the disease’s pathogenicity(43), rather than part of a compensatory effect. Quantifying this distinction will be difficult, but doing so would massively improve the utility of this type of predictor, especially for translational purposes.

Additionally, our redundancy tests are not comprehensive, and even the inhibitory drug predictions might be overshadowed by some configuration of other predictive tools. We have also previously mentioned that our accuracy metric for relationship prediction does not account for the possibility that there is no relationship between two entities, and instead assumes that there is either support or opposition(12). Furthermore, although we have tested modifications to the signature-matching pipeline, we cannot claim any aspect of our pipeline to be optimal. If we simply adopt modifications as we find that they improve performance, we run the risk of overfitting our methodology to the gold-standard upon which we test it—so future work will include exploring alternatives systematically but prudently.

In fact, we cannot claim that our methods are not already overfitted. Despite our efforts to minimize bias via cross-validation and multiple-hypothesis correction, we have used Reactome and DGIdb as a gold standard before(12), and we were not blinded to them. The only definitive way to prevent this type of overfitting is a prospective study, where we freeze our predictions and use either subsequent literature or a screen of our own to validate them. This would also demonstrate SNACKKSS’ ability to facilitate novel discoveries.

There are also two minor caveats to mention: first, we have operated at the sample level, normalizing each experimental sample to its controls(21). One could instead operate at the experiment level, by grouping control and treatment samples with RummaGEO’s K-means clustering (15), calculating differential expression with DESeq2(50), and collapsing experiments with a random-effects meta-analysis(51, 52). We call this a minor caveat because, in contrast to the modifications that we tested, this is simply another way to collapse the same data into one hypothesis, which should only make a substantial difference if one of the two methods is severely dysfunctional. These slower but more-intricate approaches could be worth testing, with some efficiency-optimization and all of the same due caution against overfitting.

Second, our metrics for precision and recall of the BERT classifiers could be overestimated, as we were not blinded to the manually curated data while we were building the input mechanism. This is mostly moot, though, because the variation in performance simply from running the models on different machines already precludes us from making any claims of the NLP’s accuracy.

For best practice in evaluating complex machine learning models, we recommend not only running the model deterministically (with any and all sources of randomness fixed to specific seeds), but also running the containerized pipeline on multiple machines to determine whether this affects performance. We have demonstrated that this difference can vary unpredictably from one task to the next. Alternatively, those who are willing to disclaim exact replicability can run a model multiple times stochastically (some will test multiple “temperatures” of randomness(53)) to determine whether the overall findings are reproducible. However, any claim that the output is frozen and replicable must be backed by an additional run on a different CPU.

As it currently stands, we have demonstrated that public RNA-Seq data, while noisy, are still sufficient to prioritize candidate drug-gene relations, and when added to one’s repertoire of predictive tools, can increase the likelihood of finding a promising regulator without notably raising the risk of a false positive. Furthermore, as more studies are released, the predictive ability of SNACKKSS will improve quadratically, and it could ultimately surpass the literature-based tools. Regardless of whether it does, we anticipate that RNA- and literature-based tools will continue to complement each other, and be better used together than alone to find pharmacologic gene regulators that could be repurposed for Mendelian disorders.

## Supplementary Material

Supplement 1

Supplement 2

## Figures and Tables

**Table 1:** Performance of non-signature-based relationship predictors.

Predictor	Positive gene-gene (test statistic, p-value, p_adj_)	Negative gene-gene (test statistic, p-value, p_adj_)	Positive drug-gene (test statistic, p-value, p_adj_)	Negative drug-gene (test statistic, p-value, p_adj_)
PARMESAN consensus	2.04, 0.0416, 1	12.5, 7.33×10^−36^, 2.05×10^−34^	2.02, 0.0433, 1	2.84, 0.00446, 0.125
PubTator3 consensus	4.00, 6.41×10^−05^, 0.00179	4.07, 4.71×10^−05^, 0.00132	4.27, 1.97×10^−05^, 0.000551	3.92, 8.71×10^−05^, 0.00244
PARMESAN indirect	−3.26, 0.00112, 0.0315	25.6, 1.61×10^−144^, 4.51×10^−143^	3.02, 0.00251, 0.0704	6.16, 7.21×10^−10^, 2.02×10^−8^
PubTator3 indirect	3.02, 0.00249, 0.0697	11.3, 1.49×10^−29^, 4.16×10^−28^	4.52, 6.27×10^−06^, 0.000176	5.55, 2.82×10^−08^, 7.89×10^−07^
PA4	−6.45, 1.12×10^−10^, 3.12×10^−9^	17.3, 1.08×10^−66^, 3.03×10^−65^	1.21, 0.225, 1	9.55, 1.36×10^−21^, 3.80×10^−20^
P3A4	−6.78, 1.22×10^−11^, 3.41×10^−10^	11.9, 1.27×10^−32^, 3.56×10^−31^	1.60, 0.110, 1	23.5, 1.29×10^−122^, 3.62×10^−121^
A4C (human)	37.5, 0, 0	−1.79, 0.0727, 1	N/A	N/A
A4C (mouse)	23.7, 6.93×10^−124^, 1.94×10^−122^	2.43, 0.0152, 0.425	N/A	N/A

We provide the log-rank test results (in the format of "test statistic, p-value, p_adj_") for the literature-based tools and the ARCHS4 gene expression correlations ("A4C") in their ability to prioritize correct regulatory relations over incorrect ones. "p_adj_" is the Bonferroni-corrected p-value, with 28 hypotheses. "Indirect" refers to linking gene-gene or drug-gene relationship consensuses from a given database to gene-gene relation consensuses from that same database, using PARMESAN's two-step prediction formula. "PA4" and "P3A4" refer to the linking of PARMESAN's and PubTator3's consensuses (respectively) to the gene expression correlations in ARCHS4. "N/A" means not applicable—expression correlations alone cannot predict drug-gene relations.

**Table 2:** Examples of beneficial and detrimental effects of incorporating SA4

Modifier	Target	Direction, per DGIdb	Source	Prediction without SA4	Confidence without SA4 (0–1)	Prediction with SA4	Confidence with SA4 (0–1)
Indisulam	GAPDH	inhibitor	GuideToPharmacology	N/A	N/A	Negative	0.989796
Dinoprostone	RHOC	activator	GuideToPharmacology	N/A	N/A	Negative	0.991453
Panobinostat	SIRT2	inhibitor	Targeted Agents in Lung Cancer	Positive	0.343348	Negative	0.98913
ORY-1001	GEM	activator	GuideToPharmacology	Positive	0.224781	Negative	0.956318

Adding a new predictive tool to one's repertoire can add valuable new information alongside unwanted noise. We provide examples of both resulting from using the SNACKKSS/ARCHS4 hybrid ("SA4") alongside the other predictive tools. Each predictor scores its prediction of a relationship's directionality, and we use all Drug-Gene Interaction Database (DGIdb) relations not involving the modifier nor the target in question to calculate the expected accuracy of the predicted relation, given the score that it received. We believe whichever model has the highest expected accuracy, and show what the prediction (and the highest expected accuracy) would have been had we included or excluded SA4. Indeed, SA4 can add both correct and incorrect relationship predictions to our repertoire, and can reverse the predicted directionality of formerly correct and incorrect predictions, meaning that we must directly measure whether the benefits outweigh the drawbacks—in other words, whether including SA4 truly improves our ability to find regulators without sacrificing accuracy.

**Table 3:** A literal and limited overview of the benefits of each predictor

	Positive gene-gene	Negative gene-gene	Positive drug-gene	Negative drug-gene
**Human A4C**	Both	Neither	Coverage	Coverage
**Mouse A4C**	Coverage	Both	Coverage	Coverage
**CMA4**	Coverage	Neither	Coverage	Coverage
**SA4**	Coverage	Neither	Coverage	Coverage
**PARMESAN consensuses**	Coverage	Neither	Accuracy	Coverage
**PARMESAN indirect hypotheses**	Coverage	Coverage	Neither	Coverage
**PA4**	Coverage	Neither	Neither	Coverage
**PubTator3 consensuses**	Coverage	Neither	Both	Coverage
**PubTator3 indirect hypotheses**	Coverage	Neither	Neither	Coverage
**P3A4**	Coverage	Neither	Coverage	Coverage

For each predictor on each task (positive and negative gene-gene and drug-gene relationship prediction), we display whether incorporating its predictions (with accuracy estimated through LOOCV) led to a larger number of predictions made (Coverage), a higher maximum achievable smoothed accuracy (Accuracy), both, or neither. The ARCHS4 correlations (Human and Mouse A4C) could not be used for drug prediction, hence the corresponding fields are labeled as "N/A". CMA4 = Connectivity Map/ARCHS4 hybrid; SA4 = SNACKKSS/ARCHS4 hybrid; PA4 = PARMESAN/ARCHS4 hybrid; P3A4 = PubTator3/ARCHS4 hybrid.

## Data Availability

Our processing pipeline is publicly available, and divided into three GitHub repositories. The first, https://github.com/coledeisseroth/SNACKKSS_NLP, contains the materials needed to train and evaluate the optimal cascade of BERT models for GEO study curation. The second, https://github.com/coledeisseroth/SNACKKSS, takes the eight trained BERT models generated from the prior repository, and runs them on all of the available study metadata in GEO, then feeds the output into our default relationship-prediction pipeline (alongside the mouse-oriented version, which uses completely different data to make predictions). The final repository, https://github.com/coledeisseroth/SNACKKSS_Eval, runs the formal comparisons that we present in this article, evaluating the predictive ability of different permutations of SNACKKSS and CMap, as well as PARMESAN and PubTator3. It takes as input the experiment files (gene_sample_controls.txt and drug_sample_controls.txt) generated from running the SNACKKSS pipeline. Our Docker images (despite not being able to replicate the output on different machines) are available upon reasonable request, and can be generated anew using the Dockerfile in each GitHub repository. The raw output of our latest SNACKKSS curation run, and the latest predictions made using those curated studies, will be available at snackkss.nrihub.org upon submission for peer review.
