# Supplementary material for: Transcriptomic data and biomedical literature synergize in finding pharmacologic gene regulators
Source: bioRxiv. 2026 Mar 14:2026.03.13.708862. Preprint. [Version 1] doi: 10.64898/2026.03.13.708862 (PMC13060807; doi:10.64898/2026.03.13.708862)
Supplement: Supplement 2 [file media-2.docx]

**Supplemental materials**

**Supplemental methods**

**Training and testing GEO-curating BERT models**

For study classification, we only use the title, summary, and overall design. For sample and control classification, we use the “characteristics”, “description”, “organism”, “source_name”, “taxid”, and “title”. For target classification, we use those fields, as well as the protocol fields, as the rest of the sample description may fail to indicate what gene was being disrupted.

GEO series IDs (GSE) that we curated were shuffled with a random seed of “2025”, and split into three equal segments, and a smaller fourth one using Bash’s “split” command, which indexed divisions using the nomenclature “xaa, xab, xac, xad”. The cross-validation consisted of training a given model on three of the segments, and testing on the fourth. For any tests involving both our manually curated dataset and CREEDS’, we would maintain the same 4-fold architecture. In other words, when we tested the CREEDS-trained model on our manually curated dataset, the trained model being tested on our “xaa” division would have seen neither our nor CREEDS’ “xaa” division.

Because these BERT models cannot interpret more than 512 tokens at a time, we *chunk* larger bodies of text by breaking them in half until the segments are readable. As a safety buffer, we set the maximum token count to 500. For study, sample, and control classification, we assign a positive label if at least one of the chunked segments was labeled as positive; for target classification, we simply extract entities from each segment through token classification. All adjacent tokens labeled as positive are merged into one extracted entity name.

For training and testing our control classifier, we only classify pairs of samples if the manually curated dataset posits that the two samples differ by exactly one perturbation, which one has and the other lacks. Because BERT models are designed to classify tokens (and, by extension, bodies of text) rather than compare them, we must introduce pairs of sample descriptions in a format that the model can readily interpret. For a given putative control sample C for a known disrupted sample D, we first align each field in C’s description to its most similar field in D’s. We then create a string consisting of the field label (e.g. “genotype:”) followed by a concatenation of all of the non-aligned text in C’s description. This allows the BERT model to pick up relevant labels such as “NT”, “DMSO”, or “vehicle” in C, while ignoring labels that are identical to those in D. We concatenate all of these strings of differences across the different fields, and then chunk and classify this concatenation as described above.

For accuracy testing, we use the following metrics: For gene-disruption study classification (the drug-study curation is evaluated the same way, but we describe these metrics in the context of gene disruptions), a true positive is a GEO series that was correctly labeled as testing a gene disruption; a false positive is a series that does not test a gene disruption but was labeled as testing one; and a false negative is a series that tests a gene disruption, but was labeled as not testing one. For gene-disruption sample classification, a true positive is a GEO sample that was correctly labeled as having a gene disrupted; a false positive is a non-gene-disrupted sample marked as having a gene disrupted; and a false negative is a gene-disrupted sample labeled as not gene-disrupted. For gene target classification, a true positive is a character index range within the sample description that indicates the disrupted gene, which was perfectly identified by the AI (in other words, it captured the complete term and nothing outside of it); a false positive is an extracted index range that does not perfectly match a range describing a targeted gene (even if it overlaps); and a false negative is an index range describing a targeted gene, which was not perfectly extracted by the AI (hence, a non-perfect overlap would constitute both a false positive and a false negative).

For gene-disruption control classification, all evaluated pairs of samples involve one with a gene disruption that the other lacks, with their sets of disrupted genes being otherwise identical; a true positive is a pair that was correctly classified as a control-perturbed pair; a false positive is a pair that was falsely classified as control-perturbed; and a false negative is a true control-perturbed pair that was not classified as such. For control classification specifically, when counting the true positives, false positives, and false negatives, we downweigh them based on the number of potential controls evaluated for each sample. In other words, if one sample has 10 potential controls, each pair classified will add 0.1 to its corresponding count. This is because in our pipeline, thousands of accurate controls ascribed to one perturbed sample will only result in us properly interpreting one perturbed sample.

This training and evaluation pipeline was run twice on an Intel Xeon Silver 4114 central processing unit (CPU) (the output we use) to test replicability within the hardware, and once on an Intel Xeon Silver 4314 CPU to test for the variability between hardware. The finalized run on the GEO metadata and downstream analyses were only run once (the pipelines under the repositories “SNACKKSS” and “SNACKKSS_Eval”, on the same Intel Xeon Silver 4114 and a different 4314, respectively). Re-running them would not be worth the processor-hours, since we already know that the exact output will not be replicable.

**CREEDS data were incorporated into our evaluation framework**

CREEDS’ manually annotated study sets had several artifacts that led to formatting issues, so we had to manually clean them. The cleaned, tab-delimited tables are in our GitHub repository, “SNACKKSS_NLP”. Additionally, we manually determined whether their descriptive study labels (i.e. not explicitly written as “KO” or “KD”) qualified as KO/KD studies. After making these adjustments, the data were suitable to feed into our training and testing pipeline, just as we did with our own dataset.

**Synonym resolution**

When evaluating the models’ performance in target classification, we only consider whether the specific text describing the target was detected, not whether we were able to map the text to the correct gene product or drug. Unique identifiers for these entities will have overlapping sets of synonyms, making it difficult to establish a ground truth for benchmarking. Furthermore, we wanted to keep our NLP evaluation pipeline static by eschewing any reliance on the ever-changing lexica.

However, entity normalization(1) is necessary in large-scale meta-analyses, so for the finalized run of SNACKKSS on the GEO metadata, we link the gene targets to their Entrez IDs and the chemicals to their PubChem substance IDs. Our normalization algorithm is as follows: we first find all potential IDs for each entity name extracted from the text. If one identified name shares an ID with a longer identified name, it will be assigned to whichever ID that longer name was assigned to. If an identified name has no extracted synonyms that are longer than itself, it is assigned to its numerically smallest ID.

**Filtering control classification data**

We use five criteria to limit the number of control classification instances. 1. The potential control must have all of the automatically identified disruptions in the perturbed sample except one. We handle this criterion differently between the accuracy testing and the finalized run: in the former, we index entities by their names alone, to minimize the susceptibility of our analysis to changing external databases. In the finalized run, however, which is already highly susceptible to this, we index genes by their Entrez IDs and chemicals by their PubChem Substances IDs (SIDs). 2. The potential control must not have any automatically identified disruptions not found in the perturbed sample (except DMSO, a common vehicle that is often not mentioned in perturbed sample descriptions). The same differences from Criterion 1 apply here. 3. The potential control must not differ from the perturbed sample in any of the fields that, in our manually curated dataset, we used to separate experiments (barring those labeled with “genotype”, “condition”, or “vector” for gene disruptions, and “exposure” or “treatment” for drugs). This filter does not apply to the accuracy tests, because that would compromise our evaluation. 4. The potential control must not differ from the perturbed sample in more than eight different description fields, which was the highest number of differing fields observed in a valid control in our manually curated dataset. This filter does not apply to the accuracy tests. 5. The GEO series containing these samples must have fewer than 10,000 comparisons to be made. This filter is solely for feasibility, as some studies can generate millions of sample pairs that need to be compared.

We run the top-performing control classifier on the first chunk of the alignment of each pair in this filtered list. Thus, for each target gene, we have a set of samples that are classified as having that gene disrupted, and for each of those samples, we have a set of appropriate control samples that do not have that specific gene disrupted. A sample with multiple gene disruptions can have multiple sets of controls, one for each disruption that it received.

**Handling samples from multiple read count datasets**

ARCHS4 collapses replicates into one GEO sample (one “GSM” identifier), whereas Recount3 and DEE2 do not collapse the replicates, and index their samples using their sequence read archive (SRA) run IDs (with “SRR” identifiers). If one perturbed sample has multiple runs, we treat each run as a separate sample with the same features. Likewise, if a control sample has multiple runs, all of its runs are taken as control samples.

All read counts are first converted to transcripts per million (TPM). For a given experimental sample with a disruption of gene A and at least two control samples, we use its controls to establish a mean and standard deviation for the TPM of each gene B. We would then calculate a z-score for the TPM of B in this disrupted sample. If a given sample has multiple disruptions, each with its own respective controls, we interpret this as two perturbed samples with different disruptions and normalize them accordingly. In other words, if Sample A is treated with Drugs X and Y, Samples B and C are treated with X, and Samples D and E are treated with Y; then we create two normalized samples from A: one treated with X and normalized to D and E; and one treated with Y and normalized to B and C.

We keep the three pre-computed read-count datasets completely separate from one another until we are calculating the consensus signature of a given gene’s disruption. When normalizing individual perturbed samples to their controls, we only use controls that are present in the same read count dataset. In other words, if Recount3 and DEE2 both have perturbed sample A, but Recount3 has controls B and C while DEE2 has controls C and D, we interpret this as two separate perturbed samples: Recount3’s Sample A (normalized to Recount3’s B and C) and DEE2’s Sample A (normalized to DEE2’s C and D).

**Connectivity Map processing**

We accessed the Connectivity Map (CMap), and downloaded their level-5 analysis. Because the data files are given in a unique binary format with the extension “.gctx” that takes a considerable amount of memory to open (the level-5 compound file took us approximately 34 gigabytes of memory), we first converted them into gzipped Unicode text files. The siginfo_beta.txt file was used to determine which collapsed, normalized samples were receiving gene-targeting shRNAs or being treated with small-molecules. For a given perturbed sample, its controls had to be in the same cell line and have the same incubation time. Dosage was not taken into consideration—this proof-of-concept, if viable, should be robust to such a caveat. For drug-treated samples, the controls had to have received a DMSO vehicle, and for shRNA-knockdown samples, any sample with the label “ctl_vector” was accepted as a control—if this method is viable, it should be robust to any variation among control vectors.

For relationship-identification purposes, our handling of CMap’s chemical and gene identifiers is slightly different from that for SNACKKSS. When calculating consensus signatures (nested Z-scores), we consider two chemical names distinct even if they likely refer to the same chemical. As an example, CMap uses compounds that it refers to as “C-646” and “C646”, which we assume to be the same (PubChem substance ID , PCSID, 85332784), but we consider them separately throughout the signature calculation and matching process. When testing accuracy and running LOOCV, we have to establish a one-to-one name-ID map, as this ensures a fair comparison to other tools and minimizes the risk of data leakage for the LOOCV test. When we do the conversion, we assign a single identifier to each named entity, and if multiple names used in CMap’s lexicon map to the same identifier, we only use the samples using the name that is found more often. We found 273 samples using “C-646” and 20 using “C646”, and DGIdb refers to the chemical as “C646”. We thus convert “C-646” to its PCSID, and ignore the “C646” experiments.

**Supplemental tables**

**Supplemental table 1: Manual study annotations**

We manually annotated 625 GEO series. The columns are as follows:

Dataset ID: The series identifier, beginning with 200.

Accession: A redundant column, the series identifier beginning with “GSE”.

Curated_by: The first name of the individual who annotated the study.

Overexpression(OE)_Knockout(KO)_Knockdown(KD)_Other-gene-modulation(OM)_Drug(D)_None(N)_Gene-and-Drug(GD): The type of experiment being done. Each distinct controlled experiment within a series gets its own row.

Sample_annotation(G=sample_id,T=Term): How we indicate control and perturbed samples in the following two columns. “G” means we list the sample accessions (GSM), separated by semicolons. T means that any samples with the indicated string in their descriptions are considered a part of that group; strings that have to both be present are separated by ampersands, and multiple strings that can indicate a sample without one another are separated by semicolons.

Control_samples: Our indication of which samples belong to the control group.

Perturbed_samples: Our indication of which samples belong to the perturbed group.

Comparison_Requirements: If this column is not empty, there are multiple experiments meeting the indicated criteria that must be considered separately, and are distinguished from one another by the field indicated (e.g. cell line). If multiple such fields are at play, they are separated by semicolons.

Input_Term: This column can be disregarded; it is a relic of an attempt to capture ChIP-Seq studies. We later concluded that our annotation schema is not sufficient to capture all types of high-throughput sequencing studies, and since we are currently only interested in RNA-Seq, we halted the effort to annotate other types.

Testing_Condition: String that must be present in the sample descriptions of both the control and the perturbed samples.

Perturbagen_terms: All strings (semicolon-separated) indicating the name of the perturbed gene or the chemical being administered.

Comments: Additional clarifications, written in plain language.

**Supplemental table 2: Corrected manual study annotations**

The modified study annotations, which we use for the pipeline. They are in the same format as Supplemental table 1. Note that the experiments previously classified as “GD” are now separated into individual experiments when possible.

**Supplemental table 3: Cross-validation split**

We used Bash’s “shuf” and “split” commands to establish our 4-fold cross-validation cohorts. In this table, we list the GEO series IDs belonging to each cohort, xaa-xad, for both our manually curated dataset SNACKKSS-MC, and CREEDS’ dataset.

**Supplemental table 4: GEO metadata NLP performance**

We provide the performance results from the NLP pipeline classifying GEO metdata. For each classification task, training each model on each manually curated corpus on each machine, we provide the number of true positives, false positives, and false negatives achieved on our SNACKKSS-MC dataset, and on that from CREEDS. For control classification, we downweigh the counts by the number of potential controls evaluated for a given disrupted sample. For example, if a given perturbed sample has 10 candidate controls, each pair will count as 0.1 cases.

**Supplemental table 5: Differential expression of targeted genes**

For gene-perturbed samples curated by SNACKKSS and Connectivity Map, we provide the number of samples where the supposedly perturbed gene increased or decreased in expression, relative to its respective controls. We show this count at baseline, and as one requires a larger expression Z-score to consider a gene differentially expressed (“Minimum expression |z-score|”). For SNACKKSS, there are three databases (ARCHS4, Recount3, and DEE2), and each one has data from two species (Human and Mouse). For Connectivity Map, we separately consider samples whose supposedly targeted gene was among the “Landmark”, “Best-inferred”, or “Inferred” genes from their assay, and we also separate samples that underwent a “knockout”, “knockdown”, or “overexpression”. At the top, we provide the total number of samples in the measured group (for example, there were 19,025 gene-perturbed samples curated by SNACKKSS with data in ARCHS4), the overall proportion of these samples that showed decreased expression (“Proportion decreased”, 81.0%), the lower and upper bounds of the binomial 95% confidence interval around this percentage (80.4-81.6%), the binomial p-value for this proportion with an expected value of 50% (“Binomial p”, 0), and the Bonferroni-corrected p-value, where SNACKKSS and Connectivity Map have 6 and 9 hypotheses, respectively (“Binomial padj”, 0).

**Supplemental table 6: Log-rank tests of DF1 signature-matching at different DEG z-score thresholds**

This table provides the log-rank test statistics for each SNACKKSS- (“SNACKKSS”) and Connectivity-Map- derived (“ConnectivityMap”) predictor’s ability to prioritize correct relations over incorrect ones. We separately test their ability to predict gene-gene (“gene”) and drug-gene (“drug”) relations, and evaluate their prioritization ability overall (“all”) and when limiting to positive (“pos”) and negative (“neg“) relationship predictions, We measure the performance at each z-score threshold (0-0.9) above which one will accept a differentially expressed gene (DEG). Our main performance metric, however, is the performance achieved after deciding the threshold through leave-one-out cross-validation (LOOCV), and for this trial, we plot the test statistic (“LOO statistic”) and the corresponding log-rank p-value (“LOO pvalue”). Since we are technically introducing 72 different relationship predictors, and the success of any of them could theoretically be considered an overall success, we run Bonferroni correction on the log-rank p-values, multiplying all of them by 72 (“LOO padj”). In addition to SNACKKSS (“default”), we display the same performance metrics for all of its permutations: requiring gene-disruption samples to have decreased expression of the supposed target (“targdown”), only using readcount data from ARCHS4 (“archs4”), and using mouse data instead of human (“mouse”). For Connectivity Map, there is also a “default” setting, and the permutations include limiting to shRNA knockdown data (“shrna_only”), including inferred gene expression levels (“inferred”), limiting to data taken from MCF7 cells (“mcf7”), and refining gene-disruption DEGs using overexpression data (“oe_corrected”). Additionally, we derive predictions from direct signature-matching (“f1_matches”), or from linking those signature matches to ARCHS4’s correlations (“archs4_correlation_linked”, i.e. SA4 and CMA4).

**Supplemental table 7: Correct and incorrect predictions from direct signature-matching-based predictors**

We display the numbers of correct and incorrect relationship predictions made by each signature-matching-based predictive tool, upon limiting the predictions to those within each score quantile. For example, in the quantile of 0.8, we only accept the top-20%-scoring predictions. For each predictor, we separately evaluate gene-gene (“Gene”) and drug-gene (“Drug”) regulatory relationships, using the manually curated databases (MCDBs) from Reactome and DGIdb (respectively) as gold standards. A “true positive” or “false positive” is a prediction of a supportive relation that, according to the MCDB, is supportive or inhibitory, respectively; likewise, a “true negative” or “false negative” is a relation predicted to be inhibitory, where the MCDB claimed it to be inhibitory or supportive, respectively. “SNACKKSS DF1” matches signatures curated by SNACKKSS, while “CMap DF1” matches consensus signatures from Connectivity Map. In addition to the “default” pipelines for each one, we test the performance of each permuted pipeline. For SNACKKSS, “ARCHS4-only” only uses read counts from ARCHS4, not from Recount3 or DEE2; “Mouse” uses mouse data instead of human, and “Target-down” only accepts gene-disruption samples if the supposedly targeted gene showed decreased expression relative to the controls. For Connectivity Map, “shRNA-only” ignores the CRISPR data, “MCF7” limits to samples from the MCF7 cell line, “OE-corrected” refines differentially expressed gene (DEG) lists using overexpression data, and “inferred” uses all inferred expression levels, rather than just the landmark genes.

**Supplemental table 8: Correct and incorrect counts for non-signature-based predictors**

This table has the same format as Supplemental table 7, but evaluates the performance of non-signature-based predictive tools. “PARMESAN consensus” and PubTator3 consensus” run PARMESAN’s consensus algorithm on relationships extracted by PARMESAN and PubTator3, respectively. “PARMESAN indirect” and “PubTator3 indirect” run PARMESAN’s indirect hypothesis formula on the relationships from PARMESAN and PubTator3, respectively. “PA4” and “P3A4” link the consensus relationships from PARMESAN and PubTator3 (respectively) to the correlations from ARCHS4 to make these predictions. “A4C” refers to predicting regulatory effects using just the coexpression matrices from ARCHS4, and we test their accuracy using either human (“Human”) or mouse (“Mouse”) data.

**Supplemental table 9: Correct and incorrect counts for predictors that link signature matches to ARCHS4’s correlations**

This table has the same format as Supplemental table 7, but evaluates the accuracy of predictions made by linking each signature-matching-based predictor to ARCHS4’s correlations. “SA4” and “CMA4” link the signature-matches from SNACKKSS and Connectivity Map (respectively) to these correlations.

**Supplemental table 10: Justification of the top-scoring inhibitory drug-gene relation from SA4**

We provide the evidence supporting the top-scoring inhibitory drug-gene relationship prediction from the SNACKKSS/ARCHS4 hybrid (SA4). The drug would have a similar or opposite signature to an intermediate gene, whose expression would have a strong correlation with that of the target gene. The columns are the intermediate gene’s Entrez ID, the DF1 score from the drug to the intermediate, the expression correlation between the intermediate and target genes, and the score for this individual link, signifying its contribution to the overall conclusion.

**Supplemental table 11: Ablation test**

We use leave-one-out cross-validation (LOOCV) to have each predictive tool estimate its own accuracy, then believe the most confident predictor regarding an unseen regulatory relationship. We display the number of correct predictions made above the lowest confidence level that yielded the desired accuracy (“Minimum accuracy”). We test this performance using 10 different predictors (or 8 for drug-gene relations) in the “Nothing” column, and after removing each predictor from the cohort. “PARMESAN consensus” and “PubTator3 consensus” remove the relationship consensuses from PARMESAN and PubTator3, respectively; “PARMESAN hypotheses” and “PubTator3 hypotheses” remove the indirect predictions from PARMESAN and PubTator3, respectively; “PARMESAN ARCHS4-linked hypotheses” and “PubTator3 ARCHS4-linked hypotheses” remove PA4 and P3A4, respectively; “Human ARCHS4 coexpression” and “Mouse ARCHS4 coexpression” remove the relationships predicted using the human and mouse (respectively) coexpression matrices from ARCHS4; and “CMap ARCHS4-linked hypotheses” and “SNACKKSS ARCHS4-linked hypotheses” remove CMA4 and SA4, respectively. We separately evaluate supportive (“Positive”) and inhibitory (“Negative”) gene-gene and drug-gene relations. A valuable contribution from a predictor is signified by inferior performance after removing it—in other words, its column has smaller correct prediction counts than the “Nothing” column. This table directly corresponds to Figure 4 and Supplemental figure 6.

**Supplemental table 12: Ablation test statistics**

For each predictor evaluated in the ablation test (Supplemental table 11), we statistically evaluate the improvement (or lack thereof) from adding each predictive tool to our repertoire. In the case of SA4, for every distinct accuracy value achieved using all predictors except SA4, we determine whether the combination of all predictors (including SA4) achieved a higher accuracy, and identified more relationships above that accuracy than we achieved without SA4. The number of accuracy values where the inclusion of SA4 successfully improved coverage is provided under “Thresholds improved”, and the number where adding SA4 did not improve our coverage above that accuracy is under “Thresholds not improved”. Likewise, we calculate the proportion of these accuracy values where the coverage improved (“Fraction improved”), after adding 1 to the denominator (“Smoothed fraction improved”), calculate a binomial p-value for whether this proportion is different from 50% (p-value), run Bonferroni correction on this p-value for 40 hypotheses (“Bonferroni-corrected p-value”), and calculate a binomial 95% confidence interval around this proportion (“Lower-“ and “Upper bound fraction improved”), and the highest smoothed accuracy achieved under the given setup (“Maximum smoothed accuracy”). We separately evaluate predictions of supportive (“+”) and inhibitory (“-“) gene-gene (“gene”) and drug-gene (“drug”) relations. The “nothing” rows are a negative control, which should have zero improvements, since it will be equal to our baseline.

**Supplemental table 13: Leave-one-out cross-validation with each predictor alone**

In the same format as our ablation test (Supplemental table 11), we display the results of using each predictor alone (rather than using all predictors except one), where through leave-one-out cross-validation, we have the predictor estimate its own accuracy and make predictions on the masked relations.

**Supplemental table 14: Inhibitory drug target coverage with and without SA4**

Since SA4 shows a clear benefit in identifying inhibitory drugs, even alongside other predictive tools, we measured the improvement in coverage in a different way. For any accuracy one is willing to accept, we measure the number of genes for which an inhibitory drug was correctly identified, when using all predictors from Supplemental table 11 (“With SA4”), or using all of them except SA4 (“Without SA4”). We also display the difference between the two (“Delta”) and the “Fold-increase” for visualization purposes. This table directly corresponds to Supplemental Figure 7.

**Supplemental figures**


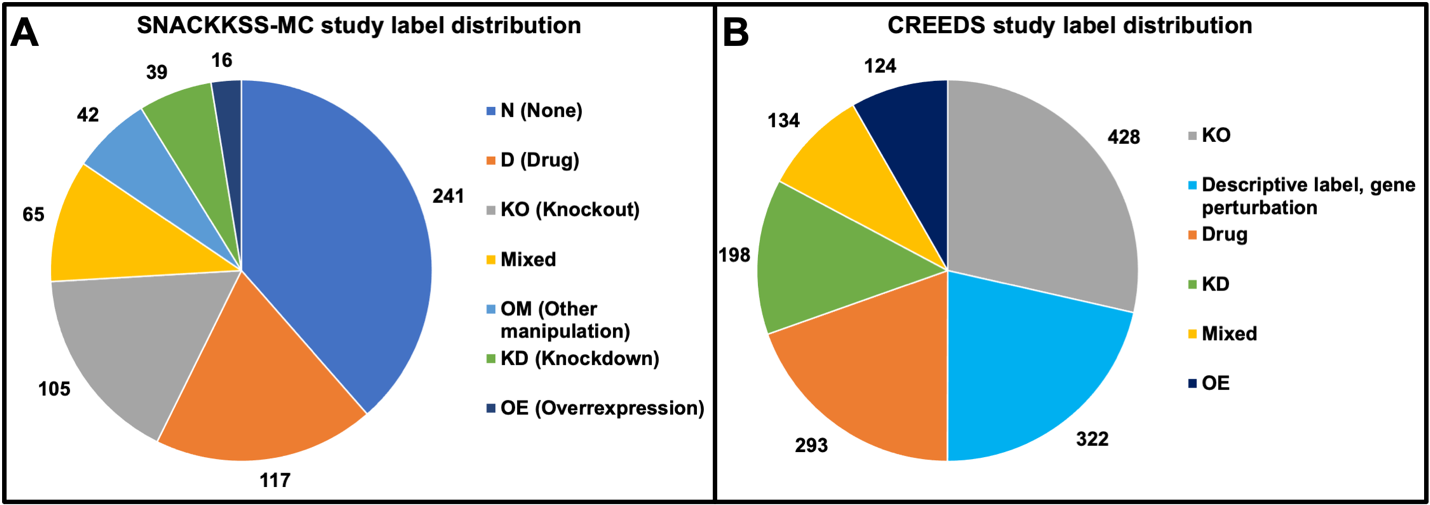


**Supplemental Figure 1: Types of GEO series observed in the manual curation**

We display the number of GEO series in our dataset, SNACKKSS-MC (A); and CREEDS’ dataset (B); labeled as containing each type of experiment. “Mixed” means that there were multiple experiment entries for this series, which did not all have the same label. “Descriptive label, gene perturbation” means that a given study from CREEDS’ single-gene cohort had a plain-text label other than “KO”, “KD”, or “OE”.


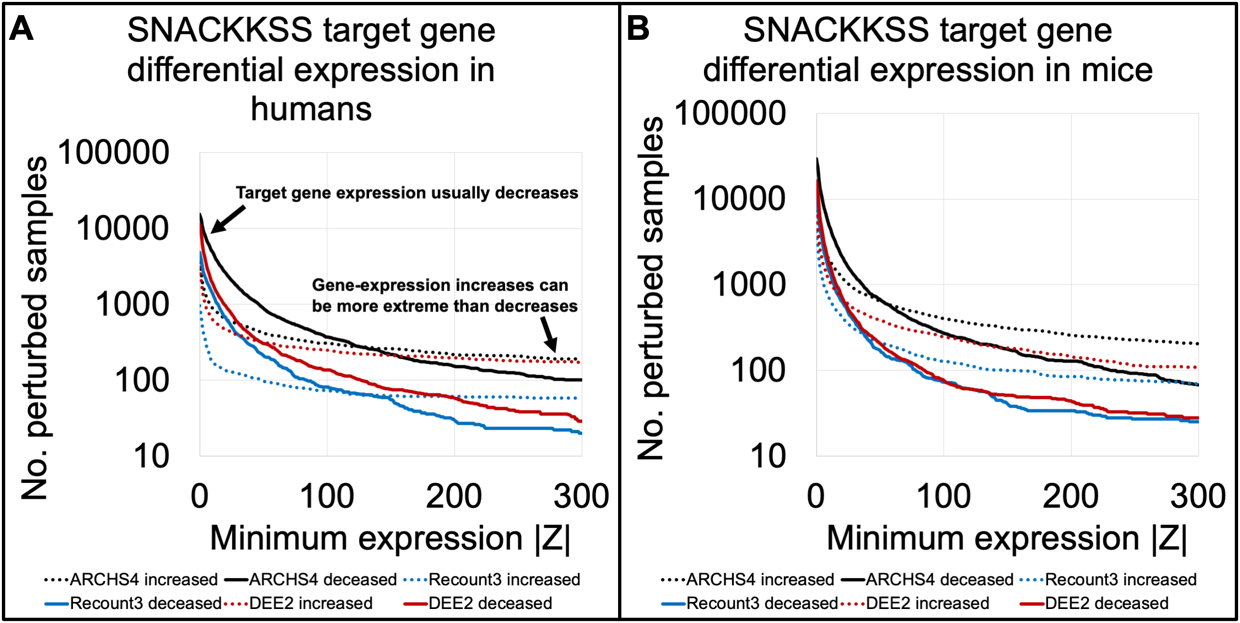


**Supplemental figure 2: Z-scores of the supposed target genes across normalized gene-disruption samples**

For all human (A) and mouse (B) read-count data taken from either ARCHS4, Recount3, or DEE2, we identify the gene-disrupted samples, and measure the z-score of the expression of the gene that was allegedly disrupted, relative to that sample’s controls (difference from the control mean divided by the standard deviation among the controls). The X axis is the minimum absolute z-score accepted, and the Y axis is the number of samples whose targeted gene had an absolute z-score above that threshold, with reduced (solid lines) or increased (dotted lines) expression of that target gene. As expected, the normalized perturbation samples predominantly showed decreased mRNA levels of their supposed KO/KD targets in all cases—except at the extremes, as the increases could be substantially stronger than the decreases. This is unsurprising, because read counts can increase to infinity, but cannot go below zero.


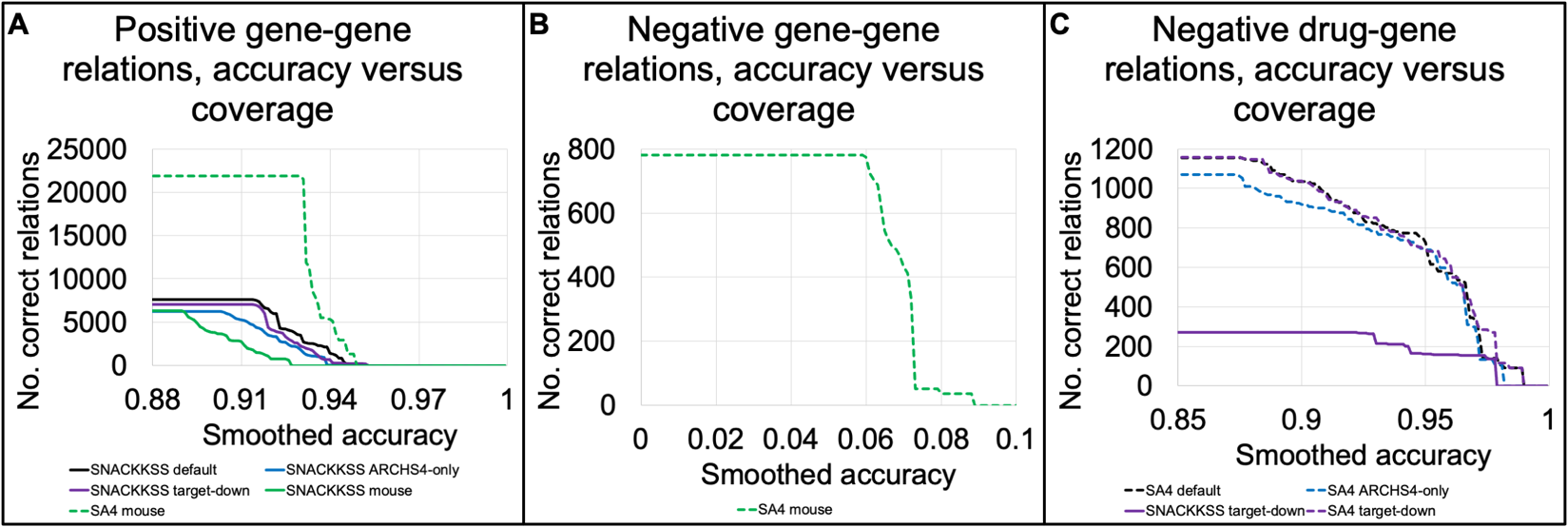


**Supplemental Figure 3: Evaluation of permutations to SNACKKSS-based predictors**

Alongside the default signature-matching approach for SNACKKSS (“SNACKKSS”) and the predictions derived from linking them to ARCHS4’s correlations (“SA4”), we test three high-level modifications to SNACKKSS and SA4 for their ability to prioritize correct regulatory relationships. The permutations require decreased expression of the supposedly disrupted gene relative to the control samples (“target-down”), eschew the use of Recount3 and DEE2 (“ARCHS4-only”), and use mouse data instead of human (“mouse”). We separately compare their performance for positive gene-gene (A), negative gene-gene (B), and negative drug-gene relations (C). We only plot a predictor if its correct predictions outlasted the incorrect ones with rising score thresholds, with an unadjusted log-rank p < 0.05. Since no setup effectively prioritized positive drug-gene relations, we do not have a panel for this task.


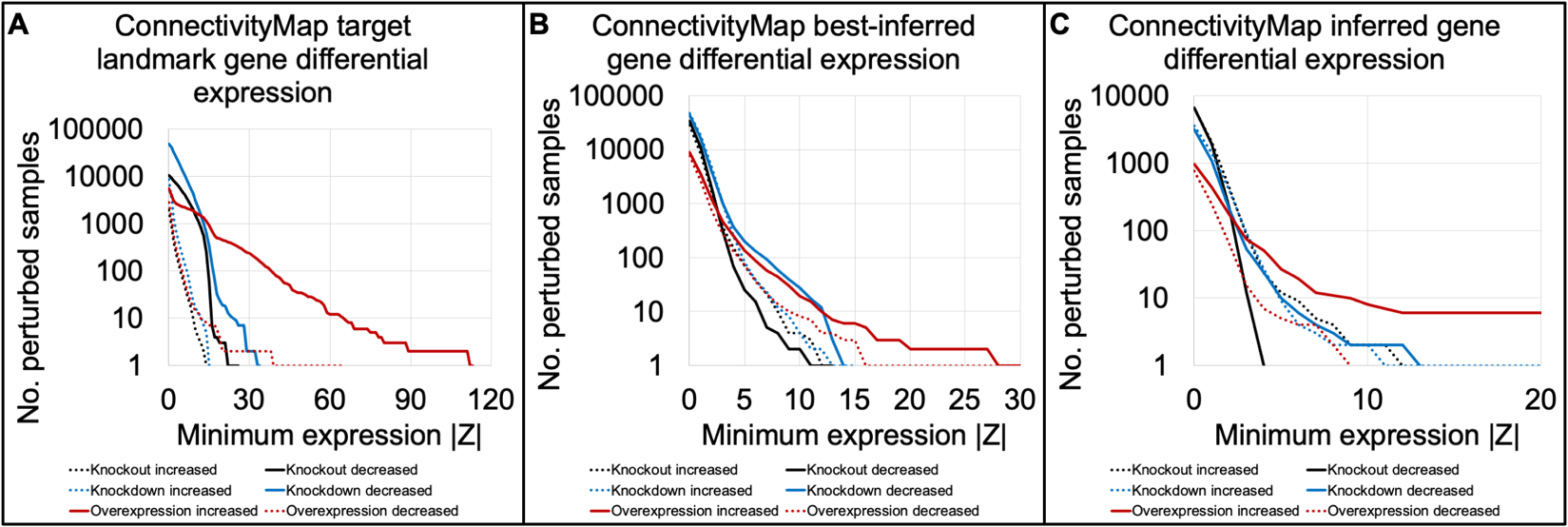


**Supplemental figure 4: Differential expression of supposedly targeted genes in the Connectivity Map**

This figure is formatted in the same way as Supplemental Figure 2, where we plot the number of samples from Connectivity Map (CMap) that, relative to their controls, had increased or decreased expression of their supposedly targeted gene. We plot this separately for shRNA-knockdown, CRISPR-knockout, and overexpression samples. Because CMap only directly measures the levels of 978 genes, we separately analyzed the “landmark” genes they directly measured (A), the “best inferred” genes whose inferred levels supposedly correlated with actual expression levels (B), and the “inferred” genes whose inferred levels supposedly did not correlate with actual expression levels (C).


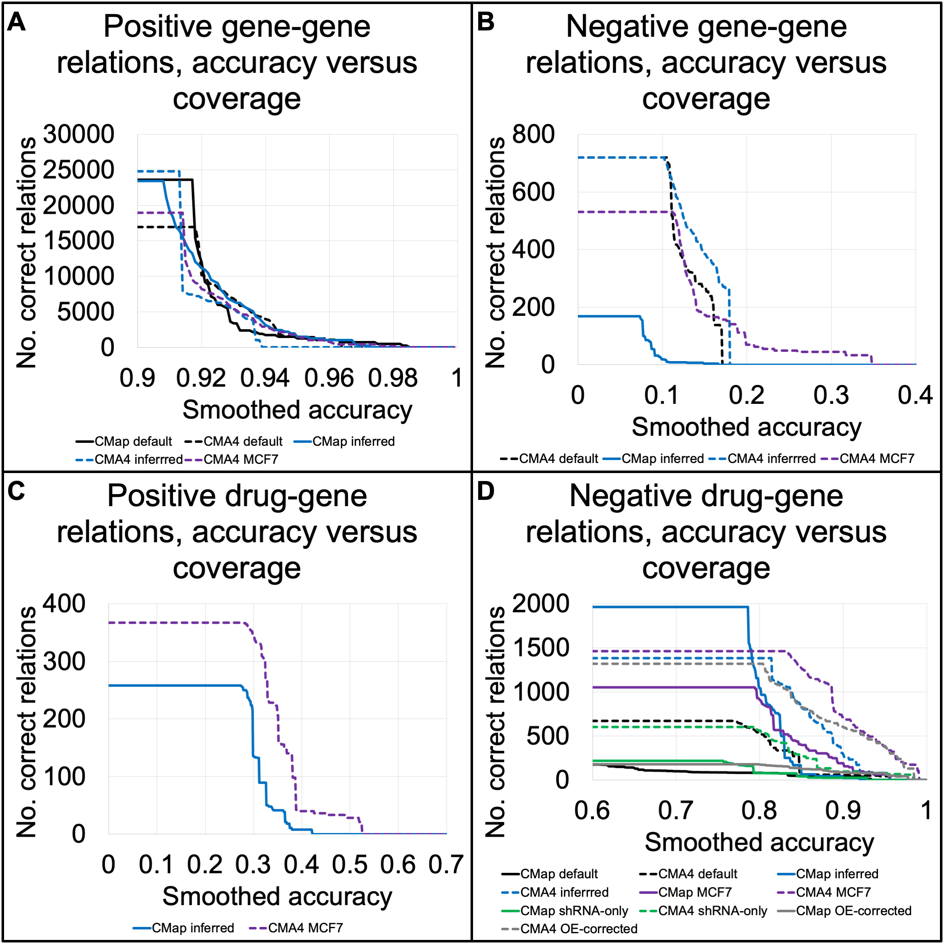


**Supplemental figure 5: Evaluation of permutations to the Connectivity-Map-based predictors**

We display the performance of matching Connectivity Map signatures (“CMap”) and linking those matches to ARCHS4’s gene expression correlations (“CMA4”, panel B) using default parameters (“default”) or one of the four permutations to the pipeline. Specifically, we limit the gene-disruption data to shRNA knockdowns (“shRNA-only”), include the inferred gene expression levels as part of the signatures (“inferred”), exclusively use data taken from MCF7 cells (“MCF7”), and limit the gene-disruption differentially expressed genes to those that went in the opposite direction upon overexpression of that gene (“OE-corrected”).

We plot the accepted accuracy against the number of correctly identified manually curated database (MCDB) relations for positive gene-gene (A), negative gene-gene (B), positive drug-gene (C), and negative drug-gene relations (D), and only display a predictor if its correct predictions outlasted the incorrect ones as the score threshold rose, with an uncorrected log-rank p < 0.05.


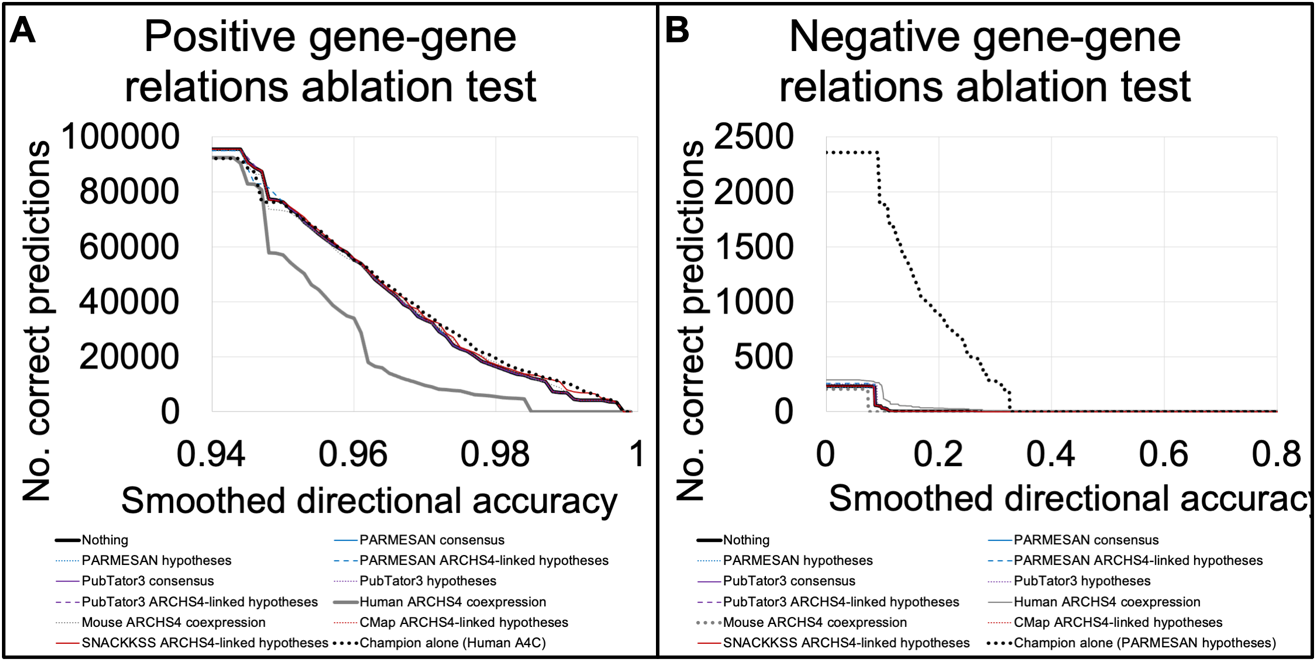


**Supplemental figure 6: Contribution of each predictor to identification of gene-gene relations**

In the same format as Figure 4, we test the performance of using all tested predictors together to identify regulatory gene-gene relations, and after removing each predictor from the ensemble, through leave-one-out cross-validation. For gene-gene relations, the ensemble did not seem to outperform the best tool by itself—ARCHS4’s human expression correlations for positive, and PARMESAN’s indirect predictions for inhibitory relations.


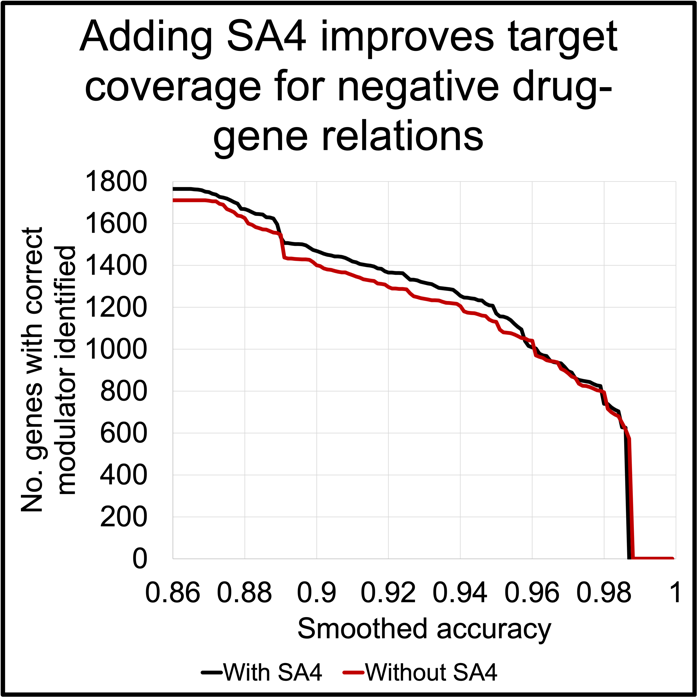


**Supplemental figure 7: Coverage improvement from adding SA4**

We plot the effect that adding SA4 to our repertoire has on the number of targets for which we can correctly identify an inhibitory drug (Y axis), for any accuracy one is willing to accept (X axis). The data plotted here are from the same LOOCV analysis used in Supplemental table 11. “With SA4” (black line) uses the same eight drug predictors from the ablation test, and “Without SA4” uses all of them except SA4, demonstrating a clear benefit in coverage upon adding this new predictor.

**Supplemental reference**

1. Wei,C.-H., Allot,A., Lai,P.-T., Leaman,R., Tian,S., Luo,L., Jin,Q., Wang,Z., Chen,Q. and Lu,Z. (2024) PubTator 3.0: an AI-powered literature resource for unlocking biomedical knowledge. *Nucleic Acids Res*, **52**, W540–W546.
